# Independent Predictors of Major Limb Amputation: A Systematic Review and Meta-Analysis of Adjusted Risk Factors

**DOI:** 10.3390/healthcare14152340

**Published:** 2026-08-01

**Authors:** Julian Deisenhofer, Maher Ghandour, Merkur Alimusaj, Andre Lunz, Burkhard Lehner, Axel Horsch

**Affiliations:** 1 Orthopedic Department, Heidelberg University, 69118 Heidelberg, Germany; julian.deisenhofer@med.uni-heidelberg.de (J.D.); merkur.alimusaj@med.uni-heidelberg.de (M.A.); andre.lunz@med.uni-heidelberg.de (A.L.); burkhard.lehner@med.uni-heidelberg.de (B.L.); axel.horsch@med.uni-heidelberg.de (A.H.); 2 Orthopedic and Traumatology Surgery Department, Bicêtre University Hospital, AP-HP, Paris-Saclay University, 94270 Le Kremlin-Bicêtre, France

**Keywords:** amputation, surgical, risk factors, lower extremity, peripheral arterial disease, diabetic foot, meta-analysis as topic

## Abstract

**Background/Objectives**: To identify independent predictors of major amputation using adjusted effect estimates. **Methods**: PubMed, Scopus, Web of Science, and Cochrane Library were searched from inception to 1 March 2024, alongside grey literature. Studies reporting adjusted predictors of major amputation (at or proximal to the wrist or ankle) were eligible. Adjusted odds ratios (aORs) reported by ≥2 studies were pooled using random-effects models. **Results:** Forty-two studies were pooled. Markers of clinical severity showed the strongest associations: critical limb ischemia (aOR = 4.60, 95% CI 4.20–5.00), leukocytosis (aOR = 3.42, 1.72–5.13), end-stage renal disease (aOR = 3.19, 2.94–3.44), and Rutherford class ≥ IIb (aOR = 3.16, 1.87–4.45). Moderate associations included chronic kidney disease (aOR = 2.22, 1.68–2.76), current smoking (aOR = 2.21, 2.13–2.28), and several comorbidities (prior bypass surgery, chronic obstructive pulmonary disease, peripheral vascular disease; aORs 1.82–1.92). Sociodemographic factors and diabetes mellitus showed smaller but consistent effects (aORs 1.19–1.64). Lower serum albumin (aOR = 0.58, 0.45–0.70) and statin use (aOR = 0.71, 0.66–0.75) reduced odds. Hemoglobin, modelled per unit increase rather than dichotomised, was unexpectedly associated with increased odds (aOR = 1.59, 1.55–1.63), most plausibly reflecting residual confounding. Heterogeneity was substantial for most predictors (I^2^ frequently > 80%). **Conclusions:** To our knowledge, this is the first meta-analysis of major amputation restricted to adjusted estimates across diverse populations and both amputation levels. Ischemic burden and systemic disease severity dominate the risk profile, whereas diabetic foot ulcer, wound depth, wound infection, coronary artery disease, and neuropathy did not retain independent significance after adjustment. Given the observational evidence base and substantial heterogeneity, these findings should inform risk stratification rather than causal inference.

## 1. Introduction

Major amputations, particularly of the lower limbs, represent a substantial global health burden and are associated with profound functional impairment, reduced quality of life, and increased mortality [1,2]. Above-knee (AKA) and below-knee amputations (BKA) are most commonly performed in patients with advanced vascular and metabolic disease and often signify failure of limb salvage despite contemporary medical and surgical interventions.

The etiology of major amputation is complex and multifactorial, encompassing sociodemographic characteristics, comorbid conditions, clinical severity, and laboratory abnormalities. Diabetes mellitus, peripheral vascular disease, and chronic kidney disease have been frequently implicated, alongside nonmodifiable factors such as age and sex and modifiable behaviors including smoking [3,4,5,6]. Despite advances in revascularization strategies and endovascular techniques, major amputations remain prevalent, particularly among patients with critical limb ischemia, chronic non-healing ulcers, and severe limb-threatening presentations [7,8,9].

Numerous studies have attempted to identify predictors of major amputation; however, reported associations have been highly variable. This inconsistency is largely attributable to differences in study design, patient populations, definitions of amputation, and—critically—the use of unadjusted or incompletely adjusted statistical models [10,11,12,13,14,15,16,17,18,19,20,21,22,23]. As a result, it remains unclear which predictors represent independent risk factors rather than proxies for disease severity or confounding comorbidity. The disagreement in the literature extends beyond imprecision to the direction of effect itself. Female sex has been reported as protective in some cohorts and as neutral in others; the reported odds ratios for diabetes mellitus span from near-null to greater than three; and body mass index, hyperglycemia, and hemoglobin have each been described, in different populations, as risk factors, as protective, and as non-significant [24,25]. Such contradictions are difficult to reconcile so long as crude and adjusted estimates are treated as interchangeable, because a factor that is strongly associated with amputation in a crude analysis may be no more than a marker of the comorbidity burden that accompanies it. In addition, evidence regarding whether predictors differ by amputation level (AKA vs. BKA) or by clinical indication remains fragmented and poorly synthesized.

Existing syntheses have not resolved these inconsistencies, and the reasons are structural rather than incidental. Previous meta-analyses of amputation risk have pooled crude and adjusted estimates together, have been confined almost entirely to diabetic foot populations, or have combined minor with major amputation into a single composite outcome [24,25,26]. Each of these choices limits inference in a specific way. Pooling crude with adjusted estimates conflates confounding with independent effect, so that the resulting summary answers neither question cleanly. Restricting the evidence base to diabetic foot cohorts leaves unrepresented the large populations in whom major amputation also occurs—patients with peripheral artery disease and chronic limb-threatening ischemia, dialysis-dependent patients, and patients undergoing amputation after trauma, burns, or acute ischemia. And treating minor and major amputation as one outcome obscures the distinction that matters most to patients, since the functional and survival consequences of a toe amputation and of an above-knee amputation are not comparable. To our knowledge, no prior review has synthesized adjusted estimates alone, across the full range of clinical populations in which major amputation occurs, or examined whether the identified predictors differ by amputation level. The present study was designed to address that gap.

Accordingly, this study had two aims: to determine which predictors of major amputation remain independently associated with the outcome once confounding has been accounted for, and to establish whether those predictors differ according to the level at which amputation is performed. A corollary aim was to identify which commonly cited risk factors lose their apparent significance after adjustment, so that risk stratification may be built upon independent associations rather than upon proxies for disease severity.

## 2. Materials and Methods

### 2.1. Study Design and Search Strategy

A systematic review and meta-analysis were conducted to identify predictors of major amputation, defined as any amputation at the level of or proximal to the wrist or the ankle. The study adhered to the Preferred Reporting Items for Systematic Reviews and Meta-Analyses (PRISMA) guidelines.

Comprehensive searches of electronic databases, including PubMed, Scopus, Cochrane Library, and Web of Science, from their inception to 1 March 2024. We also searched the Grey Literature (the first 200 records of Google Scholar), as per guidelines [27]. The search terms combined synonyms and Medical Subject Headings (MeSH) terms related to “major amputation,” “above-knee amputation,” “below-knee amputation,” and determinants of risk factors like “predictors”, “regression”, “odds ratio”, and determinant”. The detailed search strategy is provided as Table S1. Additional studies were identified through hand-searching references of included studies and relevant reviews in addition to the “similar articles” function on PubMed [28].

### 2.2. Inclusion and Exclusion Criteria

Studies were eligible for inclusion if they:

- Investigated predictors of major amputation, with explicit or implicit clarification that major (rather than minor) amputation was the outcome of interest;
- Reported adjusted effect estimates (adjusted odds ratios or data permitting their calculation);
- Provided sufficient methodological detail to permit inclusion in quantitative synthesis;
- Were peer-reviewed original research articles with accessible full texts.

Studies were excluded if they:

- Did not distinguish between minor and major amputations or failed to stratify results by amputation extent;
- Reported outcomes using incompatible effect measures (e.g., correlation coefficients only, hazard ratios without convertible data);
- Were non-primary research articles (reviews, Editorials, commentaries);
- Lacked sufficient numerical data for synthesis.

No language restrictions were applied; non-English studies were translated where necessary.

The restriction to adjusted odds ratios was deliberate and reflects the comparability of the effect measure rather than any judgement about study quality. Odds ratios, risk ratios, and hazard ratios are not interchangeable quantities. A hazard ratio describes an instantaneous rate over a period of follow-up and is therefore dependent both on the length of that follow-up and on competing risks—most importantly death, which is common in this population and which competes directly with amputation, since a patient who dies can no longer undergo one. An odds ratio, by contrast, describes cumulative odds at a fixed point. Pooling the two would combine estimates that are not estimating the same parameter, and converting between them requires assumptions—proportional hazards, a constant baseline risk, negligible censoring—that cannot be verified from published summary data. We therefore prioritized internal comparability of the pooled estimate over the volume of evidence available, accepting that this reduces the number of eligible studies.

### 2.3. Article Selection Process

Following removal of duplicate records using EndNote 21 (Clarivate Analytics, Philadelphia, PA, USA), two reviewers independently screened titles and abstracts for eligibility. Full-text review was subsequently performed to confirm inclusion based on the predefined criteria. Disagreements were resolved through discussion, with consultation of a third reviewer when consensus could not be reached.

### 2.4. Data Extraction

Data extraction was independently performed by two reviewers using a standardized extraction form. Extracted data included study characteristics (author, year, country, and study design), population characteristics, sample size, demographic variables (age and sex), amputation level, and adjusted effect estimate with corresponding confidence intervals. Given the heterogeneity of predictors reported in the literature, extracted variables were prespecified and categorized into five domains: sociodemographic characteristics, comorbid conditions, laboratory parameters, medication-related variables, and clinical examination findings. For reporting and synthesis purposes, quantitative analyses were restricted to predictors and subgroups reported by at least two studies. Discrepancies in data extraction were resolved by consensus.

### 2.5. Methodological Quality Assessment

The quality of included studies was assessed using the Newcastle Ottawa Scale (NOS) tool for assessing the quality of cohort and case–control studies. This evaluation focused on the adequacy of study design, execution, and reporting, including the potential for bias in selection, performance, detection, attrition, and reporting. Each study was given an overall rating of good, fair, or poor quality according to the number of stars given to each of the assessed domains (selection “total of 4 stars”, comparability “total of 2 stars”, and outcome/exposure “total of 3 stars”) [29]. For cross-sectional studies, the adapted NOS was used with the same overall scoring (out of 9 stars) [30]. We recognize that the AXIS and Joanna Briggs Institute instruments are also advocated for cross-sectional designs. However, the adapted NOS was retained because it permitted a single, internally consistent nine-star framework to be applied across the cohort, case–control, and cross-sectional studies that together constituted the observational evidence base, avoiding a synthesis in which quality was expressed on mutually non-comparable scales.

### 2.6. Data Synthesis

All statistical analyses were performed using Stata version 18.0 (StataCorp LLC, College Station, TX, USA). Quantitative synthesis was restricted to adjusted effect estimates to evaluate independent associations after accounting for confounding. Unadjusted (crude) estimates were excluded from all pooled analyses.

Meta-analyses were conducted when at least two studies reported adjusted odds ratios for the same predictor. Adjusted odds ratios with corresponding 95% confidence intervals were pooled using random-effects models to account for anticipated clinical and methodological heterogeneity. A list of predictors of major amputation not eligible for meta-analysis is presented in Table S2. Statistical heterogeneity was assessed using Cochran’s Q test and quantified with the I^2^ statistic, with values exceeding 75% considered indicative of substantial heterogeneity and values exceeding 90% considered considerable. A strategy for handling heterogeneity above this threshold was specified in advance, since a threshold without a corresponding course of action is of little use. Where I^2^ exceeded 75%, we (i) retained the random-effects model rather than reverting to a fixed-effect model, the latter being inappropriate precisely when between-study variance is large; (ii) interrogated the source of the heterogeneity through the prespecified subgroup analyses by amputation level and clinical indication, and through leave-one-out sensitivity analysis, in order to establish whether it arose from a single aberrant study or from genuine diversity across the evidence base; (iii) where heterogeneity remained unexplained after these steps, downgraded the interpretation of the pooled estimate accordingly, treating it as a directional summary of association rather than as a precise effect size suitable for individual risk prediction; and (iv) declined to interpret the confidence interval around a highly heterogeneous pooled estimate as though it described the range of effects to be expected in a new population, since under substantial between-study variance—and particularly where very large registry studies dominate the weighting—that interval is narrow by construction and conveys a precision the underlying evidence does not support [31]. Pooled estimates derived from only two studies were regarded as exploratory throughout.

#### Subgroup and Sensitivity Analyses

Prespecified subgroup analyses were performed to explore potential sources of heterogeneity. These analyses were stratified by level of amputation (above-knee versus below-knee), by population or clinical indication (e.g., critical limb ischemia, diabetic foot populations, dialysis-dependent cohorts), and by overall versus specific subgroups when sufficient data were available. Subgroup analyses were conducted only when at least two studies were available per subgroup. Differences between subgroups were evaluated using interaction testing, with a two-sided *p* value < 0.05 considered statistically significant. Sensitivity analyses were performed to assess the robustness of pooled estimates, including leave-one-out analyses where applicable.

Publication bias was assessed using Egger’s regression test and visual inspection of funnel plots when at least ten studies were available for a given predictor. When evidence of asymmetry was detected, the trim-and-fill method was applied to estimate the potential impact of missing studies on pooled effect sizes [32]. The ten-study threshold represents a deliberate methodological constraint rather than an omission. Regression-based asymmetry tests are severely underpowered when fewer than ten studies contribute; applied to smaller pools, they yield unstable estimates and, more damagingly, frequent false reassurance, and this problem is compounded when between-study heterogeneity is high [33]. Applying Egger’s test to a pool of three or four studies would therefore have produced a number without producing evidence. As a consequence, only four predictors—age, male sex, diabetes mellitus, and peripheral artery disease—met the threshold, and small-study effects could not be formally excluded for any of the remainder. This constraint is most consequential for those predictors whose pooled effects are large but rest upon few and comparatively small studies, among them chronic obstructive pulmonary disease, peripheral vascular disease, leukocytosis, and Rutherford class ≥ IIb, where the selective non-publication of null findings would inflate the pooled estimate and would remain undetectable with the data available.

## 3. Results

### 3.1. Literature Search Results

**Figure 1:**
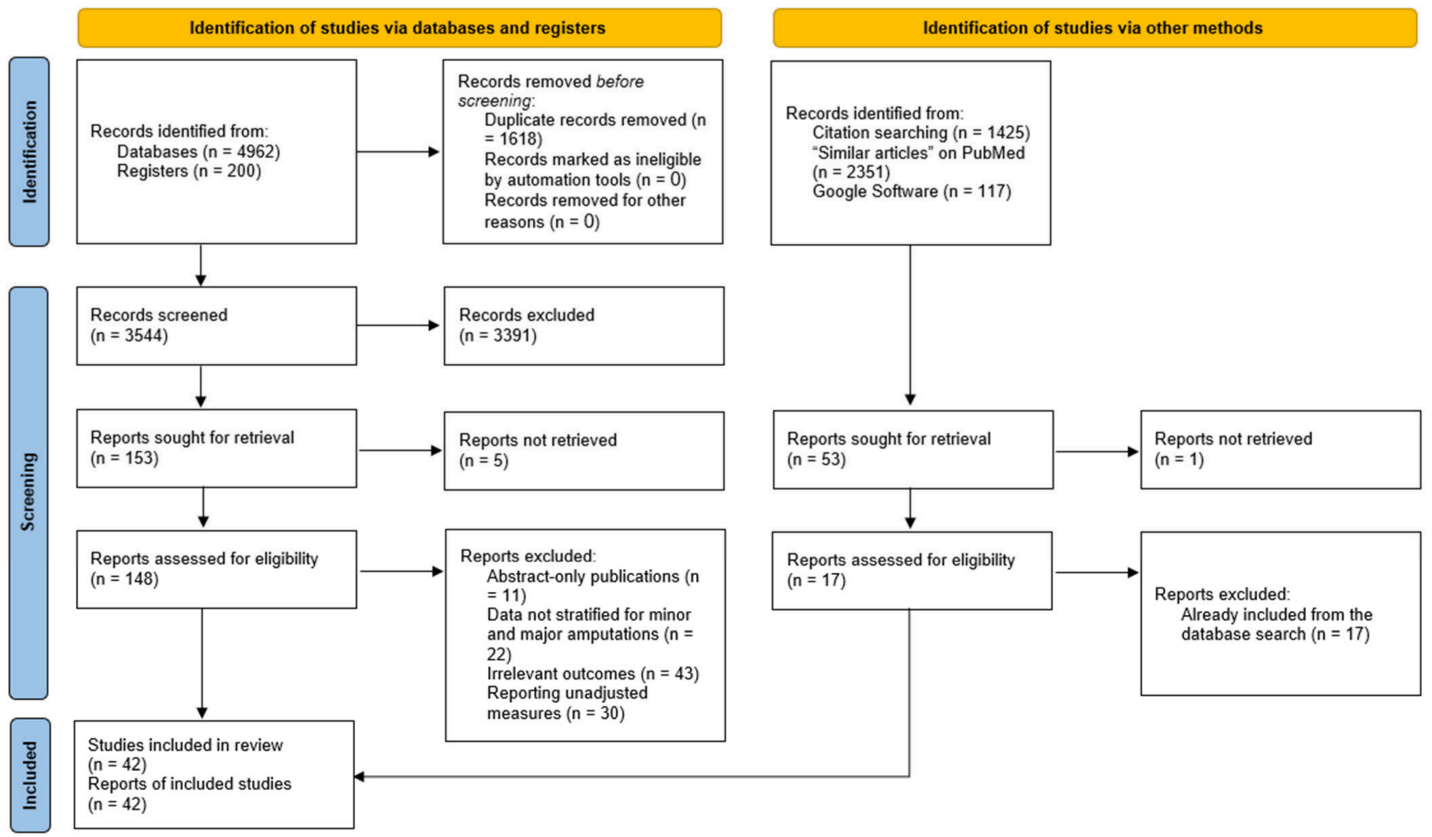
PRISMA flow diagram showing the results of the systematic literature search.

Our literature search was conducted across various databases and registers, identifying 4962 records (Figure 1). Additionally, 200 records were located through registers, resulting in an initial total of 5162 records. After removing 1618 duplicate records, 3544 records were screened. Of these, 3391 were excluded and 153 reports were sought for retrieval. Following retrieval efforts, 148 reports were assessed for eligibility, leading to the inclusion of 42 studies in the systematic review [10,11,13,15,16,17,18,19,21,22,34,35,36,37,38,39,40,41,42,43,44,45,46,47,48,49,50,51,52,53,54,55,56,57,58,59,60,61,62,63,64,65]. Articles were excluded for the following reasons: abstract-only publications (n = 11), studies including a mix of major and minor amputation cases; however, they did not provide data separately for major amputation (n = 22), studies reporting crude association measures (unadjusted OR, n = 30), and studies reporting irrelevant outcomes (n = 43). Irrelevant outcomes included predictors of mortality (n = 23), readmission (n = 17), surgical site infection (n = 15), re-amputation (n = 11), and complications (n = 10) following major amputation.

Concurrently, an alternative search method was employed, involving citation searching and examining “similar articles” on PubMed and Google Software. This method initially identified 2351 records. After screening, 53 reports were sought for retrieval, and 17 reports were assessed for eligibility. All reports assessed via this method were excluded, as they were duplicates already included from the database search. Because this alternative method yielded no additional eligible records, the final evidence base comprised the 42 studies identified through the database search, each of which contributed at least one adjusted effect estimate to the quantitative synthesis (Figure 1).

### 3.2. Characteristics of Included Studies

A total of 42 studies were included in the quantitative synthesis, together reporting 55,191 major amputations (Table 1). The included studies were predominantly observational and spanned 20 countries.

Regarding geographic origin, the largest proportion of studies was conducted in the United States (n = 13, 30.9%). Additional contributions were reported from Japan (n = 4, 9.5%), Taiwan (n = 3, 7.1%), Turkey (n = 3, 7.1%), France (n = 2, 4.8%), Malaysia (n = 2, 4.8%), Sweden (n = 2, 4.8%), and the Netherlands (n = 2, 4.8%). Single studies (each 2.4%) originated from Chile, Denmark, Fiji, Germany, Iran, Italy, Lebanon, Poland, the Republic of Korea, and South Africa.

In terms of study design, retrospective cohort studies predominated, accounting for 31 studies (73.8%). This was followed by prospective cohort studies (n = 5, 11.9%), cross-sectional studies (n = 3, 7.1%), randomized controlled trials (n = 2, 4.8%), and case–control studies (n = 1, 2.4%). Overall, the evidence base was largely observational, with randomized data contributing to a limited subset of predictors.

The included studies evaluated heterogeneous patient populations, most commonly involving individuals with peripheral artery disease, critical limb ischemia or chronic limb-threatening ischemia, diabetes mellitus and diabetic foot disease, and renal dysfunction, including dialysis-dependent populations. Several studies focused on specific high-risk cohorts, such as patients undergoing revascularization procedures, hemodialysis, or management of advanced ischemic limb disease, whereas others analyzed large registry-based or nationwide datasets.

### 3.3. Risk of Bias of Included Studies

The summary of the risk of bias of included studies, stratified by the study design is provided in Table 2. All of the three cross-sectional studies had good overall quality (low risk). Among the 36 included cohort studies, 14 were of fair quality and the remaining 22 were of good overall quality. The one case–control study had good quality. In terms of randomized trials, all of them had low risk of bias.

Heterogeneity was substantial across most pooled analyses and is central to the interpretation of every estimate reported below. Among the predictors eligible for meta-analysis, I^2^ exceeded 75% for 23 and exceeded 95% for nine, including diabetes mellitus (99.0%), dialysis (99.7%), peripheral artery disease (99.5%), hemoglobin (99.5%), critical limb ischemia (99.6%), and platelet count (100%). The prespecified subgroup analyses did not materially reduce it: within the above-knee and below-knee strata, I^2^ for diabetes mellitus remained 99.8% and 99.7% respectively, and for dialysis 99.8%. Leave-one-out analysis likewise left the direction and significance of most pooled estimates unchanged, indicating that this heterogeneity does not arise from one aberrant study but from genuine diversity in patient populations, adjustment sets, outcome definitions, and follow-up duration across the evidence base. Two consequences follow, and they apply throughout the results that follow. Pooled estimates for these predictors should be read as evidence that an association exists and in which direction it runs, not as precise quantifications of its magnitude. And the confidence intervals surrounding them—narrow because several contributing studies are very large registry analyses that dominate the weighting—convey a precision that the underlying evidence does not possess.

All subgroup analyses should be regarded as exploratory. Although stratification by amputation level and by clinical indication was prespecified, the number of studies contributing to each stratum was frequently one or two, for which no heterogeneity statistic can be computed and no pooling has in fact taken place. The interaction tests reported in those tables were not adjusted for multiplicity; with more than sixty such comparisons performed, several statistically significant subgroup differences would be expected by chance alone. Subgroup differences described below are therefore hypothesis-generating and require confirmation in adequately powered analyses.

### 3.4. Sociodemographic Predictors of Major Amputation

**Figure 2:**
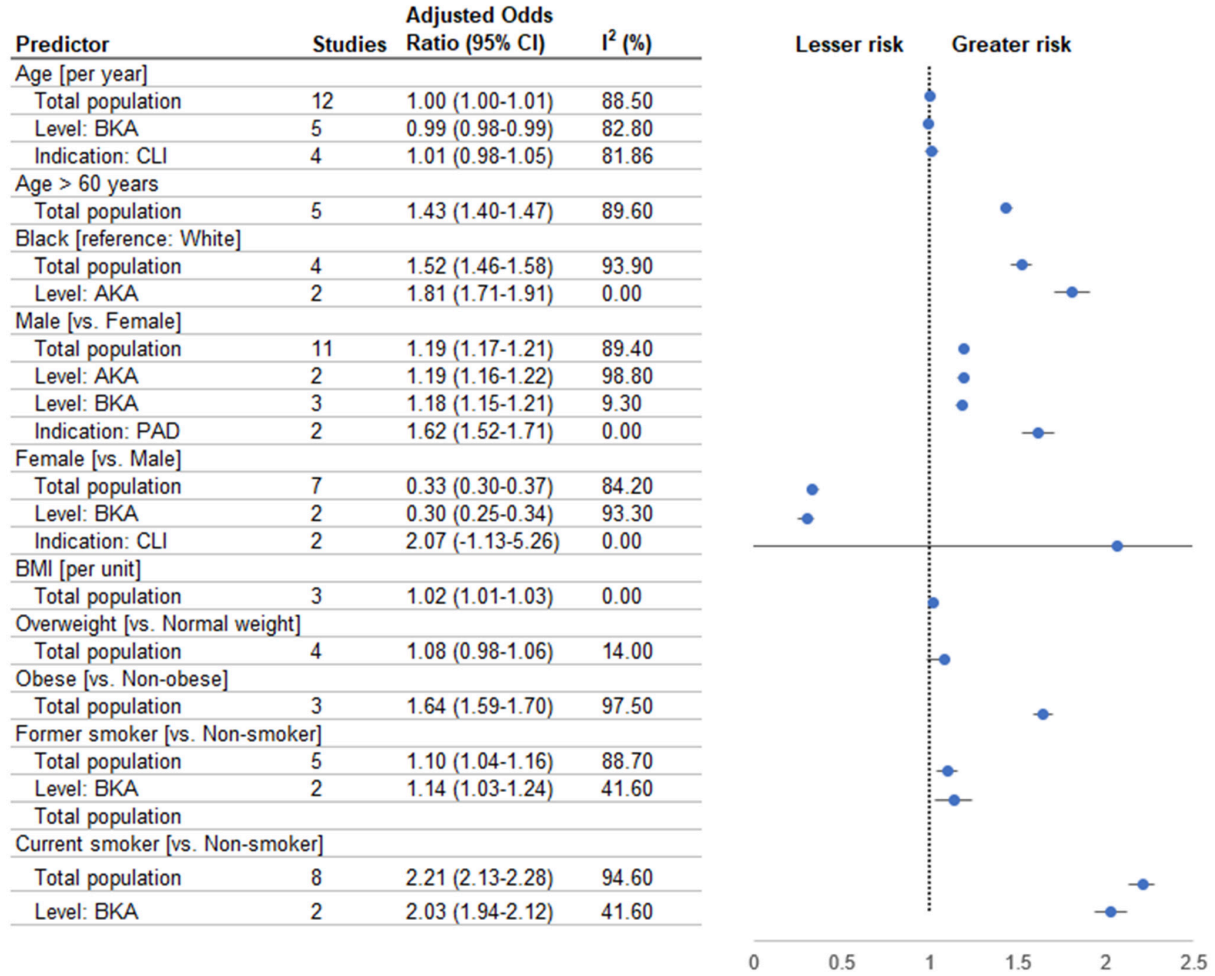
Forest plot showing the results of the pooled meta-analyses of sociodemographic predictors of major amputation in the adjusted model.

In adjusted analyses (Table 3 and Table 4, Figure 2), several sociodemographic variables were significantly associated with major amputation, with effect sizes varying by subgroup. Age (continuous) was not independently associated with major amputation in the total population (aOR = 1.00, 95% CI 1.00–1.01; I^2^ = 88.5%), the BKA subgroup (aOR = 0.99, 95% CI 0.98–0.99; I^2^ = 82.8%), or among patients with CLI (aOR = 1.01, 95% CI 0.98–1.05; I^2^ = 81.9%). In contrast, age > 60 years was associated with a higher risk of major amputation in the total population (aOR = 1.43, 95% CI 1.40–1.47; I^2^ = 89.6%). The discrepancy between these two results is one of scale rather than of substance: modelled continuously, age carries essentially no effect per additional year, whereas dichotomised at 60 years it carries a 43% relative increase in odds. Only the dichotomised estimate is clinically interpretable, and the null continuous estimate should not be read as evidence that age is unimportant.

Black race (vs White) was associated with increased odds of major amputation in the total population (aOR = 1.52, 95% CI 1.46–1.58; I^2^ = 93.9%), with a stronger and highly consistent association in the AKA subgroup (aOR = 1.81, 95% CI 1.71–1.91; I^2^ = 0%). Male sex (vs. female) was associated with increased risk in the total population (aOR = 1.19, 95% CI 1.17–1.21; I^2^ = 89.4%), and similarly in the AKA (aOR = 1.19, 95% CI 1.16–1.22; I^2^ = 98.8%) and BKA (aOR = 1.18, 95% CI 1.15–1.21; I^2^ = 9.3%) subgroups. In PAD-specific cohorts, male sex showed a stronger association (aOR = 1.62, 95% CI 1.52–1.71; I^2^ = 0%). Conversely, female sex was consistently protective in the total population (aOR = 0.33, 95% CI 0.30–0.37; I^2^ = 84.2%), the BKA subgroup (aOR = 0.30, 95% CI 0.25–0.34; I^2^ = 93.3%), and patients with CLI (aOR = 0.27, 95% CI 0.13–0.56; I^2^ = 0%).

BMI per unit increase was associated with a statistically significant but clinically negligible increase in amputation risk (aOR = 1.02, 95% CI 1.01–1.03; I^2^ = 0%); an odds ratio of this size becomes meaningful only across a wide span of BMI values and has no bearing on the assessment of an individual patient. Overweight status was not independently associated with major amputation, whereas obesity was associated with increased odds (aOR = 1.64, 95% CI 1.59–1.70; I^2^ = 97.5%). Regarding smoking, former smoking was associated with increased risk in the total population (aOR = 1.10, 95% CI 1.04–1.16; I^2^ = 88.7%) and in the BKA subgroup (aOR = 1.14, 95% CI 1.03–1.24; I^2^ = 41.6%). Current smoking demonstrated a strong association in the total population (aOR = 2.21, 95% CI 2.13–2.28; I^2^ = 94.6%) and in the BKA subgroup (aOR = 2.03, 95% CI 1.94–2.12; I^2^ = 41.6%).

### 3.5. Comorbid Predictors of Major Amputation

**Figure 3:**
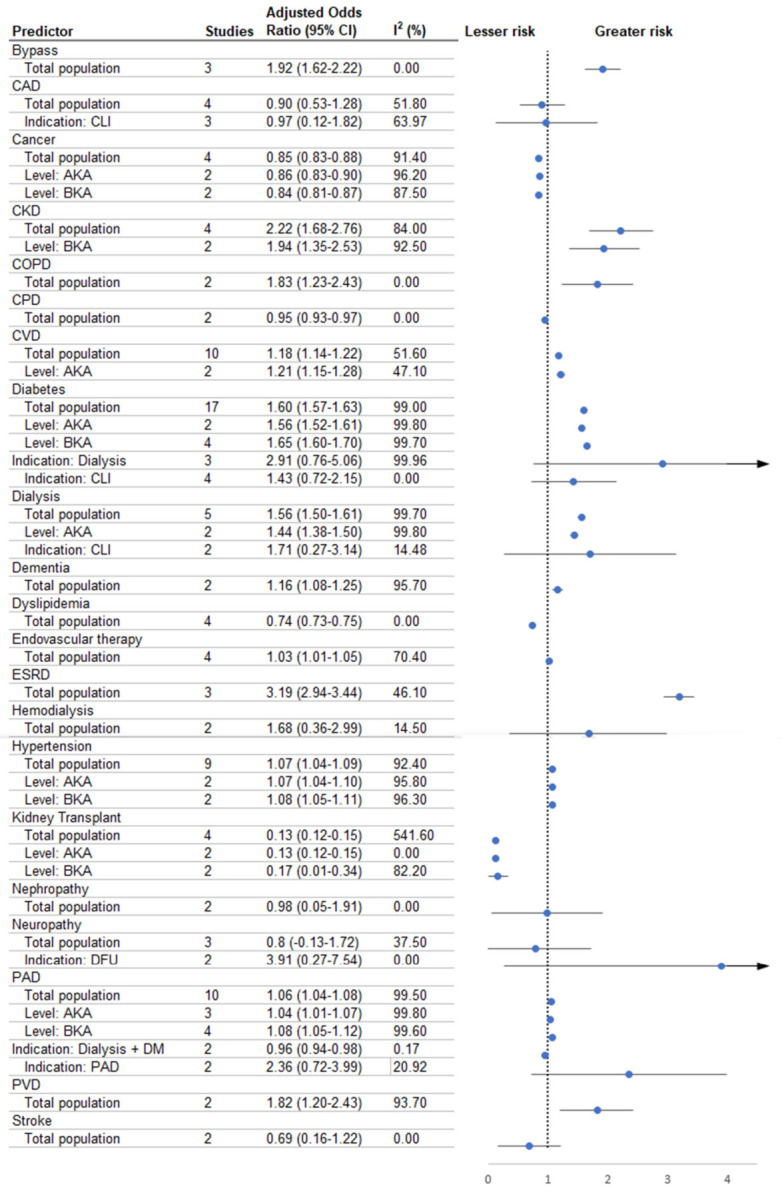
Forest plot showing the results of the pooled meta-analyses of comorbid predictors of major amputation in the adjusted model.

Several comorbid conditions were independently associated with major amputation (Table 3 and Table 5, Figure 3). A prior bypass procedure was associated with increased risk (aOR = 1.92, 95% CI 1.62–2.22; I^2^ = 0%). CKD was strongly associated with major amputation in the total population (aOR = 2.22, 95% CI 1.68–2.76; I^2^ = 84.0%) and in the BKA subgroup (aOR = 1.94, 95% CI 1.35–2.53; I^2^ = 92.5%). ESRD showed one of the largest effect sizes (aOR = 3.19, 95% CI 2.94–3.44; I^2^ = 46.1%), while dialysis was also associated with increased risk in the total population (aOR = 1.56, 95% CI 1.50–1.61; I^2^ = 99.7%) and in the AKA subgroup (aOR = 1.44, 95% CI 1.38–1.50; I^2^ = 99.8%). Diabetes mellitus was consistently associated with major amputation across analyses: total population (aOR = 1.60, 95% CI 1.57–1.63; I^2^ = 99.0%), AKA (aOR = 1.56, 95% CI 1.52–1.61; I^2^ = 99.8%), and BKA (aOR = 1.65, 95% CI 1.60–1.70; I^2^ = 99.7%).

Other comorbidities associated with increased risk included COPD (aOR = 1.83, 95% CI 1.23–2.43; I^2^ = 0%), CVD (aOR = 1.18, 95% CI 1.14–1.22; I^2^ = 51.6%; AKA: aOR = 1.21, 95% CI 1.15–1.28), PAD (aOR = 1.06, 95% CI 1.04–1.08; I^2^ = 99.5%), and PVD (aOR = 1.82, 95% CI 1.20–2.43; I^2^ = 93.7%). Hypertension was associated with a statistically significant but clinically slight increase in risk (aOR = 1.07, 95% CI 1.04–1.09; I^2^ = 92.4%). The estimates for PAD and hypertension warrant particular caution: both are significant only because very large registry cohorts dominate the pooled weighting, both carry I^2^ values above 90%, and neither effect size is of a magnitude that would alter the management of an individual patient. Conversely, kidney transplantation was strongly protective (aOR = 0.13, 95% CI 0.12–0.15), consistently across AKA and BKA subgroups. Cancer (aOR = 0.85, 95% CI 0.83–0.88) and dyslipidemia (aOR = 0.74, 95% CI 0.73–0.75) were also associated with reduced risk. CAD, stroke, nephropathy, and neuropathy were not independently associated with major amputation in adjusted analyses, except for neuropathy in DFU-specific populations.

### 3.6. Laboratory Predictors of Major Amputation

**Figure 4:**
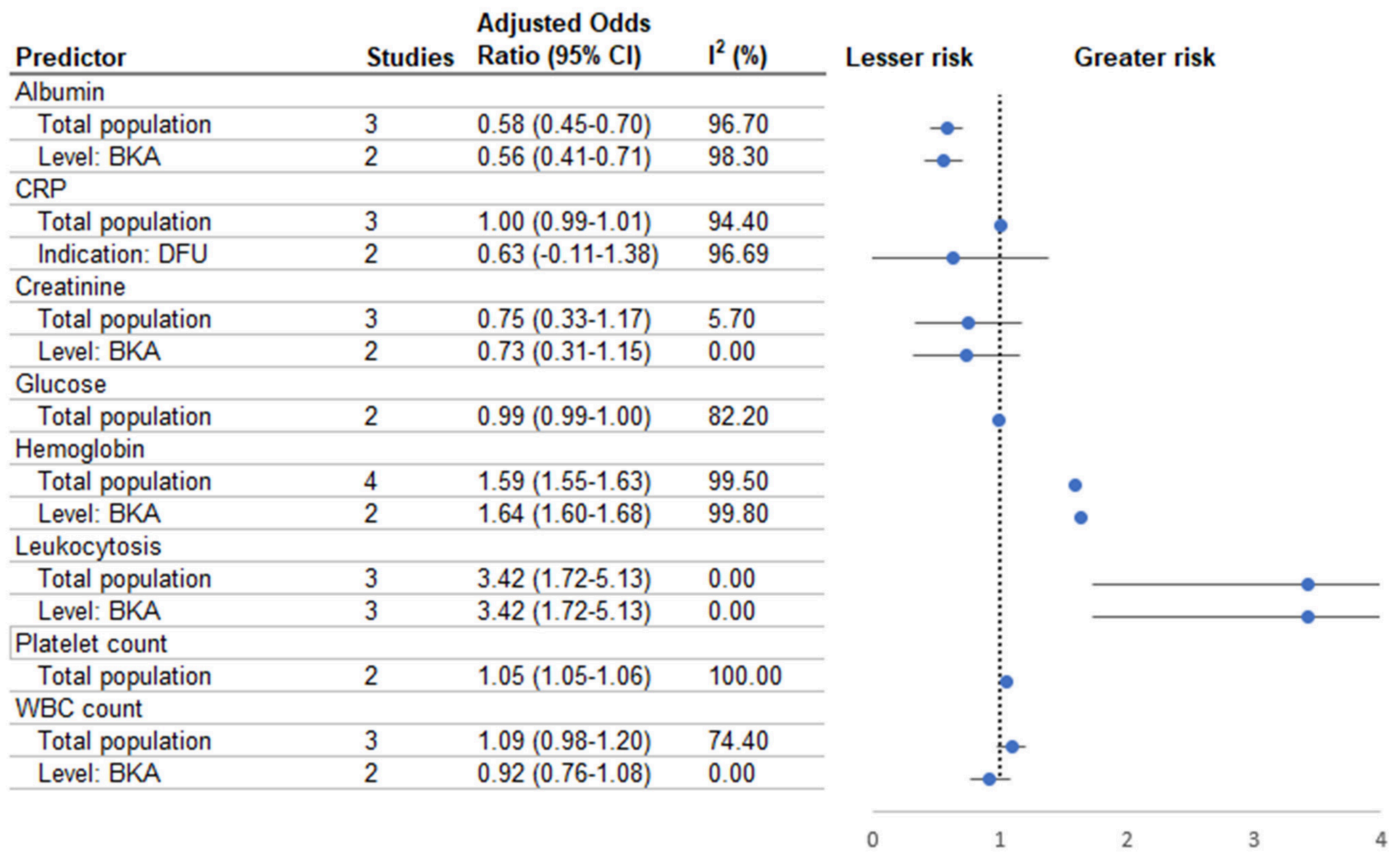
Forest plot showing the results of the pooled meta-analyses of laboratory predictors of major amputation in the adjusted model.

Several laboratory markers were independently associated with major amputation (Table 3 and Table 6, Figure 4). Among laboratory variables, lower albumin levels were associated with increased risk in the total population (aOR = 0.58, 95% CI 0.45–0.70; I^2^ = 96.7%) and in the BKA subgroup (aOR = 0.56, 95% CI 0.41–0.71; I^2^ = 98.3%). Hemoglobin, modelled in every contributing study as a continuous variable per unit (g/dL) increase rather than dichotomized at an anemia threshold, was positively associated with major amputation in the total population (aOR = 1.59, 95% CI 1.55–1.63; I^2^ = 99.5%) and in the BKA subgroup (aOR = 1.64, 95% CI 1.60–1.68; I^2^ = 99.8%). Higher hemoglobin was thus associated with higher, not lower, odds of amputation—the opposite of what the clinical literature on anemia would predict. This estimate is not stable: the direction of effect reversed in the pooled BKA–AKA stratum (aOR = 0.82, 95% CI 0.54–1.10), heterogeneity was near-total, and only four studies contributed. Anemia, analyzed separately as a binary exposure, was not eligible for meta-analysis. The finding is examined critically in Section 4.3 and should not be read as a clinical signal. Leukocytosis showed a strong and consistent association (aOR = 3.42, 95% CI 1.72–5.13; I^2^ = 0%), including in the BKA subgroup. CRP, creatinine, glucose, platelet count, and WBC count were not independently associated with major amputation after adjustment.

### 3.7. Medication-Related Predictors of Major Amputation

**Figure 5:**
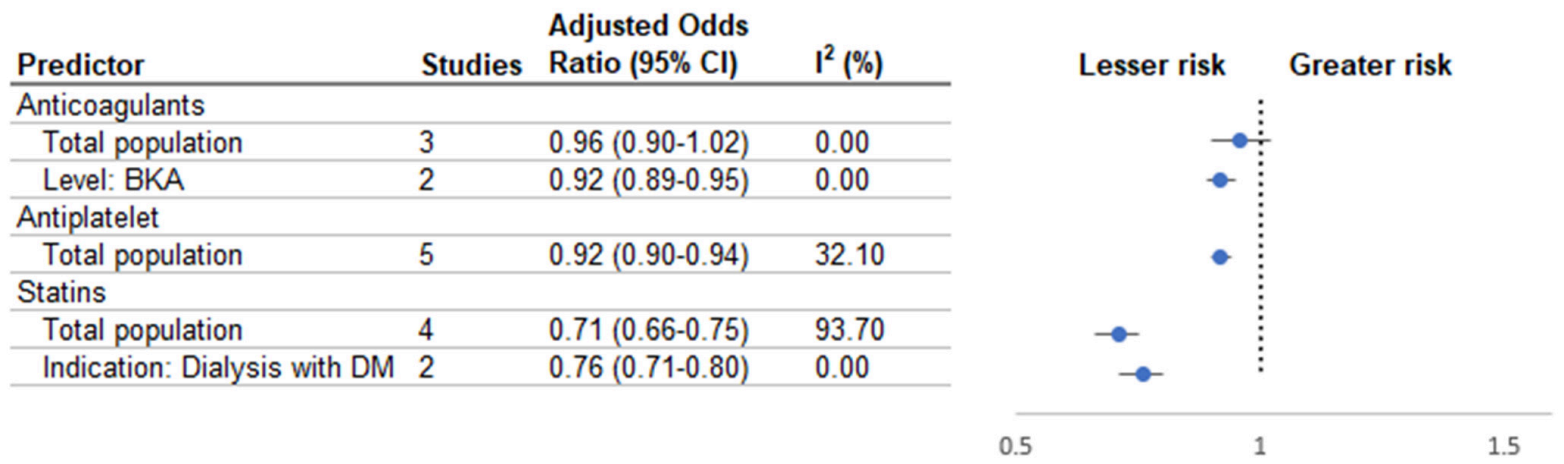
Forest plot showing the results of the pooled meta-analyses of medication predictors of major amputation in the adjusted model.

Several medications were independently associated with major amputation (Table 3 and Table 7, Figure 5). Statin use was consistently associated with reduced odds of major amputation in the total population (aOR = 0.71, 95% CI 0.66–0.75; I^2^ = 93.7%) and among patients with dialysis and diabetes (aOR = 0.76, 95% CI 0.71–0.80; I^2^ = 0%). Antiplatelet therapy was associated with a modest protective effect (aOR = 0.92, 95% CI 0.90–0.94; I^2^ = 32.1%). Anticoagulant therapy was not independently associated with major amputation.

### 3.8. Clinical Examination Predictors of Major Amputation

**Figure 6:**
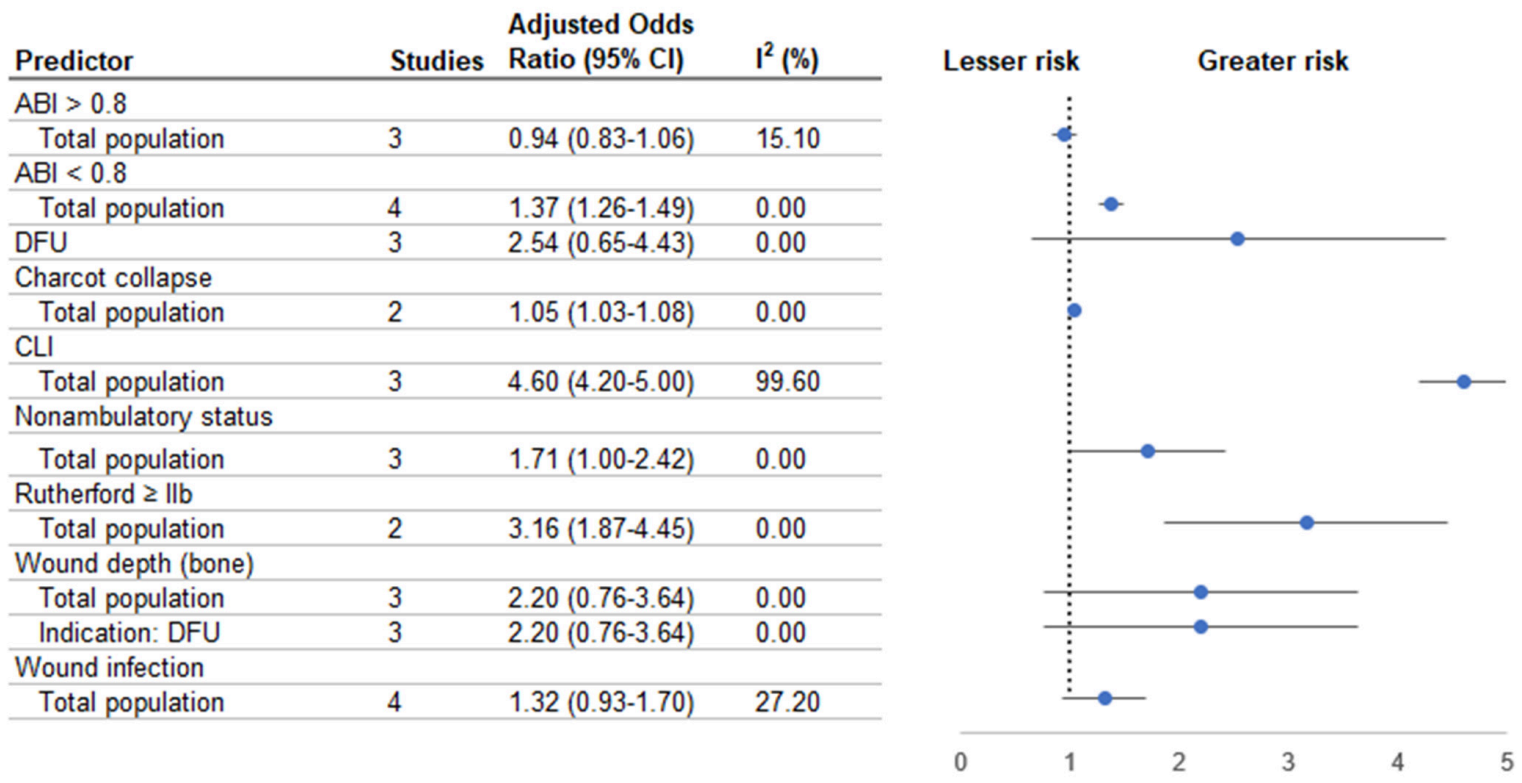
Forest plot showing the results of the pooled meta-analyses of clinical predictors of major amputation in the adjusted model.

Several clinical findings were independently associated with major amputation (Table 3 and Table 8, Figure 6). An ABI < 0.8 was significantly associated with major amputation (aOR = 1.37, 95% CI 1.26–1.49; I^2^ = 0%), whereas ABI > 0.8 was not. Markers of disease severity demonstrated the strongest associations, including CLI (aOR = 4.60, 95% CI 4.20–5.00; I^2^ = 99.6%) and Rutherford class ≥ IIb (aOR = 3.16, 95% CI 1.87–4.45; I^2^ = 0%). Non-ambulatory status was also associated with increased risk (aOR = 1.71, 95% CI 1.00–2.42). Charcot collapse showed a statistically significant but clinically trivial association (aOR = 1.05, 95% CI 1.03–1.08). Distinguishing between these findings matters: an odds ratio of 4.60 for critical limb ischemia identifies a patient group in whom limb loss is a realistic near-term prospect, whereas an odds ratio of 1.05 for Charcot collapse describes an association that is real but of no practical consequence for individual risk assessment. In contrast, DFU presence, wound depth involving bone, and wound infection were not independently associated with major amputation in adjusted analyses.

## 4. Discussion

This systematic review and meta-analysis examined predictors of major amputation using adjusted effect estimates alone. By pooling data from 42 studies, we sought to establish which associations persist once confounding has been accounted for and which do not. Three findings structure the interpretation that follows. First, the strongest and most consistent predictors were markers of ischemic and systemic disease severity rather than local wound characteristics. Second, several predictors routinely cited in the clinical literature—diabetic foot ulcer, wound depth, wound infection, coronary artery disease, and neuropathy—did not retain independent significance. Third, a small number of associations ran counter to expectation, and these we examine critically rather than accommodate. Throughout, it should be borne in mind that the evidence base is almost entirely observational and that heterogeneity was substantial for most predictors; the estimates discussed below therefore describe association rather than causation, and their magnitudes should be treated as approximate.

### 4.1. Sociodemographic Predictors

Among sociodemographic variables, age > 60 years (aOR = 1.43, 95% CI 1.40–1.47), male sex (aOR = 1.19, 95% CI 1.17–1.21), and Black race (aOR = 1.52, 95% CI 1.46–1.58) were independently associated with major amputation. The magnitudes differ considerably, and the difference is clinically consequential: the effect of male sex is modest and would not, in isolation, alter management, whereas the effect attached to Black race is comparable in size to that of diabetes mellitus. The latter association aligns with prospective evidence in patients with diabetes, in which ethnicity remained independently associated with amputation after adjustment for clinical factors [66], and with a substantial body of work documenting that Black, Hispanic, and American Indian patients in the United States undergo amputation at higher rates than White patients [67]. The mechanism is unlikely to be biological. Mediation analysis of a statewide bypass registry has shown that Black patients present more often with chronic limb-threatening ischemia, and that this later presentation—rather than race itself—accounts for their excess odds of amputation [68]. The association we report is therefore best understood as a marker of delayed access to vascular care and of the socioeconomic determinants that produce it. Its persistence after adjustment in the contributing studies most probably reflects the incomplete measurement of those determinants rather than any residual biological effect, and it should be read as an indictment of care pathways rather than as a patient characteristic.

Current smoking was among the strongest sociodemographic predictors (aOR = 2.21, 95% CI 2.13–2.28), while former smoking carried a far smaller excess risk (aOR = 1.10, 95% CI 1.04–1.16). The gradient between the two is the more informative observation, since it is consistent with a modifiable, exposure-dependent effect rather than a fixed trait, and it identifies smoking cessation as one of the few genuinely actionable targets to emerge from this synthesis. Earlier clinical work has reported an association between smoking and more proximal amputation levels, reinforcing its role as a determinant of both amputation risk and its severity [69]. Two caveats apply. Smoking status was ascertained by self-report in most contributing studies, and former smokers are systematically misclassified where quit dates are not recorded, which would tend to attenuate the apparent contrast between the two categories. Heterogeneity was also high for both estimates (I^2^ = 94.6% and 88.7%), so the point estimates should be read as directional.

### 4.2. Comorbidities

Comorbid conditions accounted for several of the strongest predictors of major amputation. End-stage renal disease (aOR = 3.19, 95% CI 2.94–3.44), chronic kidney disease (aOR = 2.22, 95% CI 1.68–2.76), diabetes mellitus (aOR = 1.60, 95% CI 1.57–1.63), and dialysis dependence (aOR = 1.56, 95% CI 1.50–1.61) were each independently associated with increased risk across populations and amputation levels. These findings are concordant with longstanding clinical observation that metabolic dysregulation, uremia, and vascular calcification impair wound healing, increase infection risk, and precipitate the failure of limb salvage [70]. It bears emphasis, however, that the estimates for diabetes and dialysis carry I^2^ values of 99.0% and 99.7%, respectively. The studies contributing to them therefore disagree almost completely about the magnitude of the effect, even while agreeing on its direction, and the pooled values are best understood as confirming that these are risk factors rather than as quantifying by how much.

Renal dysfunction showed some of the largest effect sizes in the present analysis, supporting prior reports of the compounded risk borne by patients with coexisting diabetes and kidney disease, in whom microvascular disease, immune dysfunction, and delayed tissue repair converge [4]. Kidney transplantation, by contrast, appeared strongly protective (aOR = 0.13, 95% CI 0.12–0.15). This estimate should not be taken at face value. Transplantation is not randomly allocated: candidates are selected for the absence of severe peripheral vascular disease, for adequate functional status, and for the capacity to tolerate immunosuppression, and patients whose limbs are already threatened by ischemia are precisely those least likely to be listed. An odds ratio of 0.13 is therefore better read as a measure of the selection that precedes transplantation than of any protection conferred by it. Improved metabolic control and better access to longitudinal care in transplant recipients may well contribute [71], but the magnitude of the observed effect far exceeds what those mechanisms could plausibly produce, and no causal claim is warranted.

Peripheral vascular disease (aOR = 1.82, 95% CI 1.20–2.43), prior bypass surgery (aOR = 1.92, 95% CI 1.62–2.22), and chronic obstructive pulmonary disease (aOR = 1.83, 95% CI 1.23–2.43) were also independently associated with major amputation. Prior bypass surgery most plausibly indexes advanced macrovascular disease rather than procedural failure per se, consistent with prospective analyses in critical limb ischemia demonstrating that disease severity, rather than intervention type alone, drives the likelihood of primary amputation over revascularization [10].

Peripheral artery disease presents an apparent paradox that merits direct comment. Despite being the single most important vascular determinant of limb loss, it registered a pooled estimate of only 1.06 (95% CI 1.04–1.08), with an I^2^ of 99.5%. The explanation is most likely one of restriction of range. PAD is near-universal in several of the contributing cohorts, and a risk factor present in almost every patient cannot discriminate between those who proceed to amputation and those who do not; where PAD was examined in populations in which it was not ubiquitous, the association was substantially stronger. The pooled estimate should therefore not be construed as evidence that peripheral arterial disease matters little. It is an artefact of the populations studied, and it illustrates why adjusted pooled estimates require inspection of the underlying cohorts rather than being read off a forest plot.

Two further associations ran counter to expectation and require scrutiny rather than accommodation. Cancer (aOR = 0.85, 95% CI 0.83–0.88) and dyslipidemia (aOR = 0.74, 95% CI 0.73–0.75) both appeared protective. Neither is biologically plausible as a protective factor for limb loss, and several non-causal explanations are considerably more likely. The first is competing risk of death: patients with malignancy have substantially higher mortality, and death removes a patient from the population at risk of amputation, so that a factor which increases mortality may appear to reduce amputation in any analysis that does not explicitly model competing risks—as none of the contributing studies did. The second is confounding by treatment: dyslipidemia is a coded diagnosis, and patients carrying that code are far more likely to be receiving statins and to be under structured cardiovascular follow-up than patients who do not, so the apparent protection conferred by the diagnosis may in substantial part be the protection conferred by its treatment. The third is collider bias, whereby conditioning on a variable influenced by both exposure and outcome—such as undergoing revascularization, or being admitted to a vascular service at all—can induce spurious inverse associations between factors that are in truth unrelated [72]. The fourth is differential ascertainment: comorbidity coding in administrative datasets is incomplete, and diagnoses are recorded more reliably in patients under regular surveillance, who are also those receiving better preventive care. We therefore do not interpret these estimates as evidence of protection, and we would caution firmly against any clinical inference being drawn from them.

### 4.3. Laboratory Markers

Among laboratory markers, hypoalbuminemia was independently associated with major amputation (aOR = 0.58, 95% CI 0.45–0.70 per unit increase in albumin, lower albumin therefore conferring higher risk). Low albumin has long been recognized as a surrogate for malnutrition, systemic inflammation, and frailty, all of which impair wound healing and recovery after limb-threatening ischemia [73], and prior work has linked poor nutritional status to postoperative wound complications following amputation [74]. Whether albumin represents a modifiable target or merely a barometer of systemic illness remains unresolved. The observational designs of the contributing studies cannot distinguish between these possibilities, and no trial has demonstrated that correcting hypoalbuminemia alters limb outcomes; the association is best regarded, for the present, as prognostic rather than causal.

Leukocytosis demonstrated one of the largest effect sizes in the laboratory domain (aOR = 3.42, 95% CI 1.72–5.13) with no detectable heterogeneity (I^2^ = 0%). The estimate nonetheless rests upon only three studies, could not be assessed for small-study effects, and carries a correspondingly wide confidence interval. Its interpretation is not straightforward. Elevated white cell counts are conventionally read as evidence of infection, yet leukocytosis performs poorly as a marker of specific local pathology in this setting: it is an unreliable indicator of osteomyelitis in the diabetic foot [68], and in the present synthesis wound infection itself did not retain independent significance. One hypothesis that would reconcile these observations is that leukocytosis in this population indexes global physiological derangement—systemic inflammation, sepsis, extensive tissue necrosis—rather than a localized infective process, and that it is this derangement, and not the white cell count as such, that drives limb loss. We highlight this explicitly as a hypothesis and not as a conclusion. Our data cannot test it, and it would need to be examined directly in studies that measure local and systemic inflammatory burden separately.

The hemoglobin result is the most counterintuitive finding of this review and warrants candid treatment rather than reconciliation. Hemoglobin was modelled as a continuous variable, per unit (g/dL) increase, in every contributing study; none dichotomised at an anemia threshold, and anemia as a binary exposure was analysed separately. The pooled estimate therefore indicates that higher hemoglobin was associated with higher odds of major amputation (aOR = 1.59, 95% CI 1.55–1.63). This is the opposite of what the clinical literature would predict. It is anemia, not polycythemia, that is the established correlate of adverse limb and survival outcomes in peripheral artery disease [37], and preoperative anemia has been associated with mortality and morbidity following infrainguinal bypass—though it is worth noting that in that series it was not associated with limb loss itself [75].

This finding should not be read as evidence that higher hemoglobin increases amputation risk, and we highlight four reasons for caution. First, the estimate is unstable. Heterogeneity was near-total (I^2^ = 99.5%) and the direction of effect was not consistent across strata: within the below-knee stratum the association was positive (aOR = 1.64, 95% CI 1.60–1.68), whereas in the pooled BKA–AKA stratum it reversed and lost significance (aOR = 0.82, 95% CI 0.54–1.10). A pooled estimate whose sign inverts between subgroups is not describing a stable underlying effect. Second, the analysis rests on four studies and is dominated by very large registry cohorts, whose narrow confidence intervals lend the summary an appearance of precision it does not merit. Third, hemoglobin is not measured on a common scale of clinical meaning across these populations: in dialysis-dependent cohorts it is a treated variable, actively targeted with erythropoiesis-stimulating agents, so that a higher value may index more intensive renal replacement therapy—and therefore more severe renal disease—rather than better haematological status. Fourth, hemoconcentration arising from dehydration or acute illness raises measured hemoglobin in precisely those patients who are most acutely unwell. Each of these mechanisms would generate a positive association in the complete absence of any causal effect. We therefore report the finding transparently but decline to interpret it as a clinical signal, and we regard it as a clear indication that hemoglobin requires analysis with explicit attention to renal replacement therapy, anemia treatment, and volume status before any conclusion can responsibly be drawn.

### 4.4. Medications

Statin therapy (aOR = 0.71, 95% CI 0.66–0.75) and antiplatelet therapy (aOR = 0.92, 95% CI 0.90–0.94) were both associated with reduced odds of major amputation. The statin estimate is consistent in direction and broadly in magnitude with a prior meta-analysis in peripheral arterial disease, in which statin use was associated with reduced amputation and higher-intensity regimens conferred greater benefit [76], and it is biologically supported by the pleiotropic effects of statins upon endothelial stabilization and inflammation. The antiplatelet estimate, by contrast, is statistically significant but clinically slight: an 8% relative reduction in odds is not, in itself, a basis for treatment decisions.

Both estimates are vulnerable to confounding by indication, and in a direction opposite to the usual concern. Patients not prescribed statins in these cohorts are disproportionately those judged too frail, too advanced, or too near the end of life to benefit, so that non-use marks poor prognosis rather than causing it. A healthy-user effect operates to similar purpose: patients who take prescribed medication reliably are also those who attend follow-up, undergo foot surveillance, and stop smoking. Causality cannot therefore be inferred from these data, and we frame the findings as consistent with, rather than as demonstrating the benefit of, guideline-concordant medical therapy in limb preservation.

### 4.5. Clinical Severity Markers and Limb Ischemia

Clinical findings reflecting ischemic severity showed the strongest associations with major amputation in this synthesis. Critical limb ischemia carried the single largest effect size of any predictor examined (aOR = 4.60, 95% CI 4.20–5.00), followed by Rutherford classification ≥ IIb (aOR = 3.16, 95% CI 1.87–4.45), non-ambulatory status (aOR = 1.71, 95% CI 1.00–2.42), and an ankle–brachial index below 0.8 (aOR = 1.37, 95% CI 1.26–1.49). These findings reinforce the central role of ischemic burden in determining limb viability and are consistent with prior epidemiologic and clinical studies of PAD and CLI [71], as well as with the emphasis the Global Vascular Guidelines place upon graded assessment of ischemia in staging the threatened limb [77]. Two points temper the conclusion. The critical limb ischemia estimate carries an I^2^ of 99.6% and derives from studies whose individual estimates range from an odds ratio of approximately 1.95 to 11.9, so the pooled value of 4.60 represents a midpoint between markedly discordant results rather than a consensus among them. The non-ambulatory status estimate has a lower confidence bound of precisely 1.00 and is therefore, at best, marginally significant; it should not be presented as an established predictor.

The association between ABI and amputation risk is supported by earlier work demonstrating that both low and falsely elevated ABI values can predict adverse outcomes in critical limb ischemia, underscoring the limitations of ABI interpretation in advanced disease [78]. Consistent with this, an ABI above 0.8 showed no independent association in our analysis. This should not be mistaken for reassurance: medial arterial calcification in diabetes and end-stage renal disease renders the index falsely elevated in precisely those patients at greatest risk, and a normal or high ABI in such patients cannot be taken to exclude critical ischemia [77]. Similarly, ischemia-related wound complications following major amputation have been well described, particularly in above-knee amputations where perfusion deficits are more pronounced [79,80].

Several commonly cited clinical variables—including diabetic foot ulcer presence, wound depth, and wound infection—did not remain independently associated with major amputation after adjustment. This challenges the assumption that local wound characteristics alone drive amputation risk and suggests that systemic disease severity and ischemic burden are the more critical determinants, consistent with prior analyses emphasizing perfusion and global health status over wound appearance [10,79]. The inference must nonetheless be advanced tentatively. Absence of a statistically significant pooled association is not evidence of absence of effect, and each of these predictors was supported by only three or four studies, leaving the analyses underpowered to detect effects of moderate size.

### 4.6. Clinical Implications and Future Research

These findings describe a risk profile driven principally by systemic comorbidity burden and ischemic severity rather than by isolated clinical or laboratory abnormalities. Three of the predictors identified are modifiable—smoking, nutritional status, and medication use—and these are the targets on which early intervention should concentrate. That several traditionally emphasised predictors did not retain independent significance argues for risk stratification built on multivariable adjusted models rather than on single factors weighed in isolation.

Several directions for future research follow directly from this synthesis. First, the field requires primary studies that report adjusted estimates with prespecified and transparently reported adjustment sets. Much of the heterogeneity encountered here is driven not by clinical diversity but by inconsistency in what was adjusted for, and that source of variation is remediable at no cost. Second, major amputation competes with death, and analyses that ignore this competition will continue to generate the kind of spurious protective association we observed for cancer; competing-risk models should become the default in this literature rather than the exception. Third, the predictors identified here should be assembled and tested as a multivariable risk model rather than being interpreted one at a time, since clinicians do not encounter risk factors in isolation. Recent machine-learning models predicting outcomes after lower-extremity endovascular revascularization demonstrate that such prediction is feasible from routinely collected preoperative data and can substantially outperform logistic regression [81], and an equivalent model for major amputation, externally validated across the diverse populations represented in this review, would be of far greater clinical utility than any individual odds ratio reported here. Fourth, the counterintuitive hemoglobin association should be interrogated directly, in cohorts in which renal replacement therapy, anemia treatment, and volume status are recorded and can be adjusted for. Finally, the modifiable predictors identified in this review—smoking, nutritional status, and statin and antiplatelet therapy—are the appropriate targets for prospective intervention, and it is striking that none has yet been tested in a trial with major amputation as a primary endpoint.

### 4.7. Strengths and Limitations

The principal strength of this review is its restriction to adjusted effect estimates. By excluding crude associations, the synthesis addresses independent rather than marginal effects, and it is this restriction that allows the negative findings—the loss of significance for wound infection, wound depth, coronary artery disease, and neuropathy—to carry meaning, since in a crude analysis such factors would almost certainly have appeared significant. Further strengths include prespecified eligibility criteria and subgroup analyses, duplicate screening and data extraction, broad international and clinical representation extending well beyond the diabetic foot populations to which previous syntheses have largely been confined, and the coverage of five predictor domains within a single analytic framework.

The limitations are substantial and set real boundaries upon what may be concluded.

Heterogeneity is foremost among them. I^2^ exceeded 75% for 23 pooled predictors and 95% for nine, and neither the prespecified subgroup analyses nor leave-one-out sensitivity testing reduced it meaningfully. Under heterogeneity of this magnitude, a random-effects pooled estimate is a weighted average of effects that genuinely differ between settings, and its confidence interval—narrowed by the inclusion of very large registry studies that dominate the weighting—conveys a precision the evidence does not possess. We have therefore framed our findings consistently as evidence of the existence and direction of associations rather than of their magnitude, and we would discourage the use of the point estimates reported here as inputs to individual risk calculators without prior external validation.

Publication bias could not be excluded for most predictors. Regression-based asymmetry tests are underpowered below ten contributing studies [33], and only four predictors met that threshold. The predictors for which this matters most are precisely those with large pooled effects resting upon few and small studies—chronic obstructive pulmonary disease, peripheral vascular disease, leukocytosis, and Rutherford class ≥ IIb among them—where selective publication of positive findings would inflate the pooled estimate and would remain undetectable with the data available.

The restriction to adjusted odds ratios, though analytically a strength, is also a limitation. Studies reporting hazard ratios or risk ratios were excluded, and these included well-conducted time-to-event analyses that had appropriately handled the competing risk of death; it is possible that the excluded evidence differs systematically from the evidence we included. Moreover, adjustment sets varied widely between the included studies and were not always fully reported, so that the word “adjusted” does not denote a uniform quantity across the analyses we pooled. Residual and unmeasured confounding therefore persists despite the restriction, and is the most plausible explanation for several of the counterintuitive findings discussed above.

Risk of bias was assessed with the Newcastle–Ottawa Scale, including its adapted form for cross-sectional studies. This afforded a consistent nine-star framework across the observational designs, but the NOS was not developed for prognostic-factor research and does not interrogate the adequacy of confounder measurement or the appropriateness of the statistical analysis with the specificity of a purpose-built instrument such as QUIPS [82]. The psychometric properties of NOS adaptations remain a matter of legitimate debate.

All subgroup analyses are exploratory. Many strata contained a single study; interaction tests were not corrected for multiplicity; and with more than sixty comparisons performed, several significant differences would be expected by chance.

Finally, the outcome itself is not uniformly defined across the evidence base. Studies varied in whether they counted amputation at any point during follow-up or within a fixed window, in whether bilateral amputations were counted once or twice, and in whether amputations performed for trauma, burns, or acute infection were pooled with those performed for chronic ischemia. Follow-up duration was inconsistently reported, and because the odds of amputation rise with observation time, studies with longer follow-up contribute systematically larger estimates. The evidence base is also overwhelmingly retrospective—31 of the 42 included studies—with only two randomized trials contributing, so channeling by indication cannot be excluded for any of the treatment-related predictors.

## 5. Conclusions

In this synthesis of adjusted effect estimates from 42 studies, major amputation was independently associated with markers of ischemic severity and systemic disease burden—critical limb ischemia, advanced Rutherford class, renal failure, diabetes mellitus, and current smoking foremost among them—while several commonly cited predictors, including diabetic foot ulcer, wound depth, wound infection, coronary artery disease, and neuropathy, did not retain significance after adjustment. Two qualifications are essential to any use of these findings. The evidence base is almost entirely observational, so these are associations and not causes. And heterogeneity was substantial for most predictors, so the pooled magnitudes are approximate and should not be transplanted into individual risk calculators without validation. Read within those bounds, the results support a model in which limb loss is driven principally by ischemic burden and global physiological reserve rather than by local wound characteristics, and they identify smoking cessation, nutritional status, and guideline-concordant medical therapy as the modifiable targets most worth testing prospectively.

## Figures and Tables

**Table 1:** Baseline characteristics of included studies reporting predictors of major amputation (n = 42).

Author (YOP)	Country	Design	Population	Level of Amputation	Amputated Limbs	Age	Gender
						Mean (SD)	(M:F)
Axley (2020) [13]	USA	RC	Patients treated for IC	AKA	128		
Abou-Zamzam (2007) [10]	USA	PC	Patients with CLI	BKA-AKA	97	66.5	48:49
Acar (2017) [11]	Turkey	RC	Patients with diabetic foot	BKA-AKA	22	65.2	18:4
Brunelli (2015) [34]	Italy	CC	Amputees admitted in a rehab hospital	BKA-AKA	91	68.3 (10.8)	71:20
Cai (2021) [35]	USA	RC	Veterans using VA services	BKA	39,237	61 (13.5)	38,516:721
				AKA			
Chen (2015) [15]	Taiwan	RC	Patients with diabetic foot	Above the ankle	98	56.9 (13.5)	75:23
Chou (2022) [16]	Taiwan	RC	Patients with DM and diabetic foot ulcer	NCD	246	66.98 (14.32)	61:27
Clegg (2023) [36]	USA	RC	Patients with DFU	BKA-AKA	243	58.9 (10.9)	166:77
Desormais (2014) [37]	France	Registry-based study	Hospitalized patients for PAD	NCD	246	-	-
Elmarsafi (2019) [38]	USA	RC	Patients who underwent osseous Charcot reconstruction	BKA-AKA	49	61.23	-
Furuyama (2018) [39]	Japan	RC	Patients with CLI who underwent infrainguinal arterial embolization	BKA-AKA	19	-	-
Genovese (2016) [40]	USA	RC	Patients who underwent endovascular therapy for ALI	NCD	399	-	-
Goodney (2010) [41]	Lebanon	Multicenter cohort	Patients who had LL bypass procedures	BKA-AKA	140	20–100	94:46
Hallström (2021) [42]	Sweden	Registry-based study (SNDR)	T1DM patients without previous amputation	NCD	1519	50.1 (12.4)	1070:449
Hamera (2015) [43]	Poland	RC	Patients with CLI treated endovascularly	BKA-AKA	32	66.5 (9.9)	23:9
Higashi (2019) [44]	Japan	RC	Diabetic patients undergoing antiplatelet therapy for PAD	BKA-AKA	27	72.3 (6.7)	17:10
Hüsers (2020) [45]	Germany	PC	DM patients with DFU	NCD	29	68.52 (12.4)	25:4
Khan (2021) [46]	Fiji	CS	T2DM patients who had LL amputation	Through or above the ankle	213	25–75	112:101
Kobayashi (2022) [47]	Japan	RC	Patients with CLTI	NCD	34	67 (10)	23:11
Kofod (2022) [48]	Denmark	CS	Diabetic patients receiving dialysis	BKA-AKA	11	-	-
Kow (2019) [49]	Malaysia	CS	Patients undergoing surgical management for DFU	BKA	21	21–90	13:8
Kurniawati (2019) [50]	Taiwan	RC	DFU patients	BKA-AKA	14	47	-
Long (2020) [51]	USA	RCT (EUCLID)	Postrandomization major amputation in patients with PAD	BKA-AKA	216	67 (17.9)	162:54
McGinigle (2014) [52]	USA	CR	Patients with PAD	AKA	7328	18–100	4323:3004
Mendes-Pinto (2021) [53]	USA	RC	Patients with CLTI	AKA	33	-	-
Miyajima (2006) [17]	Japan	RC	Patients with diabetic foot gangrene	BKA-AKA	45	20–75	37:8
Mohapatra (2018) [18]	USA	RC	Patients with ischemic foot wounds and PAD	NCD	71	-	-
Mtshali (2020) [54]	South Africa	RC	Patients with DM-related LL amputation	BKA-AKA	99	18–60	43:56
Namgoong (2016) [55]	South Korea	RC	Patients with diabetic foot	BKA-AKA	28	-	-
Niakan (2020) [56]	Iran	PC	Patients with DFU	Above the ankle	18	-	-
O’Hare (2003) [57]	USA	Database-based study	Patients undergoing hemodialysis	BKA-AKA	1317	-	937:380
Ozbeyaz (2022) [58]	Turkey	RC	Patient with below-knee PAD	BKA	37	71 (10.3)	25:12
Pickwell (2015) [19]	The Netherlands	PC	Patients with infected DFU	NCD	33	66.4	22:11
Rosedi (2022) [59]	Malaysia	RC	DFU patients	NCD	66	20–80	23:43
Schneider (2018) [60]	France	PC (SURDIAGENE)	T2DM patients	Above the ankle	31	-	-
Seo (2023) [61]	Republic of Korea	Registry-based (KNHIS)	Patients with ESRD	AKA	363	58.88 (11.09)	245:118
				BKA	2113	58.07 (10.58)	1515:598
Smith (2016) [21]	USA	RC	Patients with bypass grafts	NCD	89	69 (11.6)	58:31
Soto (2013) [22]	Chile	RC	Severe burn patients	Above or below the elbow	64	43.7 (18.8)	51:13
Spreen (2018) [62]	The Netherlands	A pooled analysis of 2 trials	Patients with CLI	Through or above the ankle	59	-	-
Taşoǧlu (2014) [63]	Turkey	RC	Patients with acute limb ischemia undergoing embolectomy	NCD	36	70.2 (8.5)	23:13
Torbjörnsson (2020) [64]	Sweden	RC	Patients with CLI	Above the ankle	178	76 (14.9)	98:80
Zhang (2020) [65]	USA	RC	Patients with acute leg compartment	BKA-AKA	52	54 (18)	35:17

YOP: year of publication; USA: United States of America; SNDR: Swedish National Diabetes Registry; RC: retrospective cohort; CS: cross-sectional; DFU: diabetic foot ulcer; PC: prospective cohort; RCT: randomized controlled trial; CC: case–control; T2DM: type 2 diabetes mellitus; T1DM: type 1 diabetes mellitus; DM: diabetes mellitus; CLI: critical limb ischemia; ESRD: end-stage renal disease; PAD: peripheral artery disease; IC: intermittent claudication; PVI: peripheral vascular intervention; CR: chart review; ALI: acute limb ischemia; CLTI: chronic limb-threatening ischemia; BKA: below-knee amputation; AKA: above-knee amputation; NCD: not clearly described; LL: lower limb; M:F: number of male to female patients; SD: standard deviation.

**Table 2:** The risk of bias of included studies stratified by the study design.

		Cross-Sectional Studies					
Author (YOP)	Nature	Selection (5 Starts)	Comparability (1 Star)	Outcome (3 Stars)	Overall Rating		
Khan (2021) [46]	Cross-sectional	5	1	3	Good		
Kofod (2022) [48]	Cross-sectional	5	1	3	Good		
Kow (2019) [49]	Cross-sectional	4	1	3	Good		
		Cohort Studies					
		Selection (4 starts)	Comparability (2 stars)	Outcome (3 stars)	Overall Rating		
Abou-Zamzam (2007) [10]	Prospective	4	2	2	Good		
Hüsers (2020) [45]	Prospective	4	1	2	Fair		
Niakan (2020) [56]	Prospective	4	2	2	Good		
Pickwell (2015) [19]	Prospective	4	2	2	Good		
Schneider (2018) [60]	Prospective	4	2	2	Good		
McGinigle (2014) [52]	Retrospective	4	1	2	Fair		
O’Hare (2003) [57]	Retrospective	4	2	2	Good		
Goodney (2010) [41]	Retrospective	4	1	2	Fair		
Axley (2020) [13]	Retrospective	4	1	2	Fair		
Acar (2017) [11]	Retrospective	4	2	2	Good		
Cai (2021) [35]	Retrospective	4	2	2	Good		
Chen (2015) [15]	Retrospective	4	1	1	Fair		
Chou (2022) [16]	Retrospective	4	1	2	Fair		
Clegg (2023) [36]	Retrospective	4	2	2	Good		
Elmarsafi (2019) [38]	Retrospective	4	1	2	Good		
Furuyama (2018) [39]	Retrospective	4	2	2	Good		
Genovese (2016) [40]	Retrospective	4	2	2	Good		
Hamera (2015) [43]	Retrospective	4	2	2	Good		
Higashi (2019) [44]	Retrospective	4	2	2	Good		
Kobayashi (2022) [47]	Retrospective	4	1	2	Fair		
Kurniawati (2019) [50]	Retrospective	4	2	2	Good		
Mendes-Pinto (2021) [53]	Retrospective	4	2	2	Good		
Miyajima (2006) [17]	Retrospective	4	1	2	Fair		
Mohapatra (2018) [18]	Retrospective	4	2	2	Good		
Mtshali (2020) [54]	Retrospective	4	1	2	Fair		
Namgoong (2016) [55]	Retrospective	4	2	2	Good		
Ozbeyaz (2022) [58]	Retrospective	4	2	2	Good		
Rosedi (2022) [59]	Retrospective	4	1	2	Fair		
Smith (2016) [21]	Retrospective	4	1	2	Fair		
Soto (2013) [22]	Retrospective	4	2	2	Good		
Taşoǧlu (2014) [63]	Retrospective	4	2	2	Good		
Torbjörnsson (2020) [64]	Retrospective	4	2	1	Fair		
Zhang (2020) [65]	Retrospective	4	2	2	Good		
Seo (2023) [61]	Retrospective	4	1	2	Fair		
Desormais (2014) [37]	Retrospective	4	2	2	Good		
Hallström (2021) [42]	Retrospective	4	1	2	Fair		
		Case–Control Studies					
		Selection (4 starts)	Comparability (2 stars)	Outcome (3 stars)	Overall Rating		
Brunelli (2015) [34]	Retrospective	4	2	2	Good		
		Randomized Controlled Trials					
		Randomization	Deviation from intended interventions	Missing outcome data	Measurement bias	Selective reporting	Overall Rating
Spreen (2018) [62]	Randomized	Low	Low	Low	Low	Low	Low
Long (2020) [51]	Randomized	Low	Low	Low	Low	Low	Low

YOP: year of publication. In terms of observational studies (case–control, cohort, and cross-sectional), the Newcastle Ottawa Scale (NOS) was used. As for randomized trials, the revised Cochrane risk of bias tool (Version 2) was implemented.

**Table 3:** A summary of the number of studies reporting predictors of major amputation in the adjusted model, along with the results of the sensitivity analysis and publication bias.

		Adjusted Model						
Category	Predictor	K	I^2^	*p*	Contributors to Heterogeneity	Sensitivity Analysis	Egger’s Test	Trim and Fill Analysis
Sociodemographic	Age	12	88.5	0.01	ND	Non-Sign.	0.09	-
	Age > 60 years	5	89.6	0.01	Amputation level	Non-Sign.	N/A	N/A
	Black	4	93.9	0.01	Amputation level	Non-Sign.	N/A	N/A
	Male	11	89.4	0.01	ND	Non-Sign.	0.17	-
	Female	7	84.2	0.01	Amputation level	Non-Sign.	N/A	N/A
	Weight	-	-	-	-	-	-	-
	BMI	3	0	0.83	N/A	N/A	N/A	N/A
	Overweight	4	14	0.32	ND	N/A	N/A	N/A
	Obese	3	97.5	0.01	ND	Non-Sign.	N/A	N/A
	Former smoker	5	88.7	0.01	Amputation level	Non-Sign.	N/A	N/A
	Current smoker	8	94.6	0.01	Amputation level	Non-Sign.	N/A	N/A
Comorbidity	Bypass	3	0	0.77	N/A	N/A	N/A	N/A
	CABG	-	-	-	-	-	-	-
	CAD	4	51.8	0.1	N/A	N/A	N/A	N/A
	Cancer	4	91.4	0.01	ND	Non-Sign.	N/A	N/A
	CKD	4	84	0.01	ND	Sign.	N/A	N/A
	COPD	2	0	0.43	N/A	N/A	N/A	N/A
	CPD	2	0	0.99	N/A	N/A	N/A	N/A
	CVD	10	51.6	0.02	Amputation level	Non-Sign.	0.19	-
	DM	17	99	0.01	Amputation level	Non-Sign.	0.02	1 study
	Dialysis	5	99.7	0.01	Amputation level	Non-Sign.	N/A	N/A
	Dementia	2	95.7	0.01	N/A	N/A	N/A	N/A
	Dyslipidemia	4	0	0.43	ND	N/A	N/A	N/A
	Endovascular therapy	4	70.4	0.1	N/A	Sign.	N/A	N/A
	ESRD	3	46.1	0.15	N/A	N/A	N/A	N/A
	GI disorder	-	-	-	-	-	-	-
	Hemodialysis	2	14.5	0.28	N/A	N/A	N/A	N/A
	Hypertension	9	92.4	0.01	Amputation level	Non-Sign.	N/A	N/A
	Hyperlipidemia	-	-	-	-	-	-	-
	History of minor amputation	-	-	-	-	-	-	-
	Kidney Transplant	4	54.16	0.1	ND	N/A	N/A	N/A
	Nephropathy	2	0	0.34	N/A	N/A	N/A	N/A
	Neuropathy	3	37.5	0.2	N/A	N/A	N/A	N/A
	PAD	10	99.5	0.01	Amputation level	Non-Sign.	0.22	-
	PCI	-	-	-	-	-	-	-
	Pulmonary disease	-	-	-	-	-	-	-
	PVD	2	93.7	0.01	N/A	N/A	N/A	N/A
	Renal disease	-	-	-	-	-	-	-
	Retinopathy	-	-	-	-	-	-	-
	Stroke	2	0	0.67	N/A	N/A	N/A	N/A
	T1DM	-	-	-	-	-	-	-
	T2DM	-	-	-	-	-	-	-
Laboratory Investigations	Albumin	3	96.7	0.01	ND	Non-Sign.	N/A	N/A
	ALT	-	-	-	-	-	-	-
	Anemia	-	-	-	-	-	-	-
	CRP	3	94.4	0.01	N/A	Sign.	N/A	N/A
	Creatinine	3	5.7	0.34	N/A	N/A	N/A	N/A
	eGFR	-	-	-	-	-	-	-
	ESR	-	-	-	-	-	-	-
	Glucose	2	82.2	0.01	N/A	N/A	N/A	N/A
	HbA1C	-	-	-	-	-	-	-
	Hemoglobin	4	99.5	0.01	ND	Non-Sign.	N/A	N/A
	LDL-C	-	-	-	-	-	-	-
	Leukocytosis	3	0	0.89	N/A	N/A	N/A	N/A
	Platelet count	2	100	0.01	N/A	N/A	N/A	N/A
	WBC count	3	74.4	0.02	Amputation level	Non-Sign.	N/A	N/A
Medications	ACE-I	-	-	-	-	-	-	-
	Anticoagulants	3	0	0.81	N/A	N/A	N/A	N/A
	Antiplatelets	5	32.1	0.21	ND	N/A	N/A	N/A
	Beta blocker	-	-	-	-	-	-	-
	Statins	4	93.7	0.01	ND	Non-Sign.	N/A	N/A
Clinical Examination	ABI > 0.8	3	15.1	0.31	N/A	N/A	N/A	N/A
	ABI < 0.8	3	0	0.41	N/A	N/A	N/A	N/A
	Charcot collapse	2	0	0.69	N/A	N/A	N/A	N/A
	CCI ≥5	-	-	-	-	-	-	-
	CLI	3	99.6	0.01	ND	Sign.	N/A	N/A
	DBP	-	-	-	-	-	-	-
	SBP	-	-	-	-	-	-	-
	Foot ulcer	-	-	-	-	-	-	-
	Preop gangrene	-	-	-	-	-	-	-
	Ischemic wound	-	-	-	-	-	-	-
	Laterality (right side)	-	-	-	-	-	-	-
	Major tissue loss	-	-	-	-	-	-	-
	Nonambulatory status	3	0	0.46	ND	Non-Sign.	N/A	N/A
	Rutherford ≥ IIb	2	0	0.56	N/A	N/A	N/A	N/A
	Wound dehiscence	-	-	-	-	-	-	-
	Wound depth (bone)	3	0	0.91	N/A	N/A	N/A	N/A
	Wound infection	4	27.2	0.25	N/A	N/A	N/A	N/A

N/A: not applicable (either due to the small sample size “in the case of publication bias” or the lack of heterogeneity “in the case of sensitivity analysis”); ND: not defined; Sign.: significant; ABI: ankle brachial index; CCI: Charlson comorbidity index; SBP: systolic blood pressure; DBP: diastolic blood pressure; WBC: white blood cell; LDL: low-density lipoprotein; CRP: C-reactive protein; DM: diabetes mellitus; T2DM: type 2 diabetes mellitus; T1DM: type 1 diabetes mellitus; PVD: peripheral vascular disorder; PCI: percutaneous coronary intervention; CABG: coronary artery bypass graft; PAD: peripheral artery disease; eGFR: estimated glomerular filtration rate; ESRD: end-stage renal disorder; CVD: cerebrovascular disorder; CPD: chronic pulmonary disorder; COPD: chronic obstructive pulmonary disorder; CAD: coronary artery disease; BMI: body mass index.

**Table 4:** A summary of sociodemographic predictors of major amputation in the adjusted model stratified by the level of amputation.

		AKA					BKA					BKA-AKA					*p*
Predictor	Model	K	OR	Low CI	High CI	I^2^ (*p*)	K	OR	Low CI	High CI	I^2^ (*p*)	K	OR	Low CI	High CI	I^2^ (*p*)	
Age	Adjusted	1	1.01	1	1.02	N/A	5	0.99	0.98	0.99	82.8 (0.01)	3	0.98	0.96	1	75.9 (0.01)	**0.001**
Age > 60 years	Adjusted	1	1.46	1.41	1.51	N/A	1	1.43	1.38	1.48	N/A	2	1.39	0.83	1.94	12.1 (0.28)	**0.001**
Black	Adjusted	2	1.81	1.71	1.91	0 (0.32)	1	1.38	1.31	1.45	N/A	1	1.35	0.81	1.89	N/A	**0.001**
BMI	Adjusted	-	-	-	-	-	1	1.02	1.01	1.03	N/A	2	1.02	0.97	1.06	0 (0.55)	0.904
Current smoker	Adjusted	1	2.95	2.79	3.11	N/A	2	2.03	1.94	2.12	41.6 (0.19)	4	1.33	0.36	2.3	0 (0.67)	**0.001**
Female	Adjusted	1	0.4	0.33	0.47	N/A	2	0.3	0.25	0.34	93.3 (0.01)	3	1.72	0.97	2.47	0 (0.67)	**0.001**
Former smoker	Adjusted	1	1.17	1.09	1.25	N/A	2	1.14	1.03	1.24	41.6 (0.19)	1	0.57	−0.05	1.19	N/A	**0.001**
Male	Adjusted	2	1.19	1.16	1.22	98.8 (0.01)	3	1.18	1.15	1.21	9.3 (0.3)	5	1.4	1.03	1.77	0 (0.49)	0.374
Obese	Adjusted	1	1.93	1.83	2.03	N/A	1	1.57	1.5	1.64	N/A	-	-	-	-	-	**0.001**
Overweight	Adjusted	1	0.99	0.94	1.04	N/A	1	1.05	0.99	1.1	N/A	1	1.36	-0.07	2.79	N/A	0.322
Weight	Adjusted	-	-	-	-	-	-	-	-	-	-	-	-	-	-	-	-

Bold values indicate statistically significant difference based on the amputation level. K: number of studies analyzed in each group; OR: odds ratio; CI: confidence interval; I^2^ (*p*): I-squared statistic measure of heterogeneity (*p* value of heterogeneity); *p*: *p*-value of the difference between examined groups; AKA: above-knee amputation; BKA: below-knee amputation; N/A: not applicable either because of an insufficient number of studies (K = 2) or because of insignificant heterogeneity (*p* > 0.05).

**Table 5:** A summary of comorbid predictors of major amputation in the adjusted model stratified by the level of amputation.

		AKA					BKA					BKA-AKA					*p*
Predictor	Model	K	OR	Low CI	High CI	I^2^ (*p*)	K	OR	Low CI	High CI	I^2^ (*p*)	K	OR	Low CI	High CI	I^2^ (*p*)	
Bypass	Adjusted	1	1.83	1.38	2.29	N/A	1	1.97	1.55	2.38	N/A	1	2.5	0.45	4.55	N/A	0.774
CABG	Adjusted	-	-	-	-	-	-	-	-	-	-	-	-	-	-	-	-
CAD	Adjusted	-	-	-	-	-	1	1.11	0.42	1.8	N/A	1	0.32	−0.29	0.93	N/A	**0.048**
Cancer	Adjusted	2	0.86	0.83	0.9	96.2 (0.01)	2	0.84	0.81	0.87	87.5 (0.01)	-	-	-	-	-	0.387
CKD	Adjusted	1	3.6	2.12	5.09	N/A	2	1.94	1.35	2.53	92.5 (0.01)	1	4.19	0.68	7.7	N/A	0.068
COPD	Adjusted																
CPD	Adjusted	1	0.95	0.92	0.98	N/A	1	0.95	0.92	0.98	N/A	-	-	-	-	-	1
CVD	Adjusted	2	1.21	1.15	1.28	47.1 (0.16)	1	1.16	1.11	1.21	N/A	6	0.98	0.69	1.27	0 (0.67)	**0.004**
Dementia	Adjusted	1	1.44	1.3	1.58	N/A	1	1.01	0.9	1.12	N/A	-	-	-	-	-	**0.001**
Dialysis	Adjusted	2	1.44	1.38	1.5	99.8 (0.01)	1	7.88	7.45	8.32	N/A	2	1.62	1.07	2.16	26.5 (0.24)	**0.001**
DM	Adjusted	2	1.56	1.52	1.61	99.8 (0.01)	4	1.65	1.6	1.7	99.7 (0.01)	9	1.84	1.47	2.21	56.5 (0.01)	**0.048**
Dyslipidemia	Adjusted	1	0.73	0.71	0.75	N/A	1	0.75	0.73	0.77	N/A	2	0.53	−0.02	1.08	0 (0.6)	0.29
EVT	Adjusted	1	1.01	0.98	1.04	N/A	1	1.04	1.01	1.06	N/A	1	3.42	−1.64	8.48	N/A	**0.017**
ESRD	Adjusted	1	3.27	3	3.54	N/A	-	-	-	-	-	1	5.3	−2.69	13.29	N/A	0.15
GI disorder	Adjusted	-	-	-	-	-	-	-	-	-	-	-	-	-	-	-	-
Hemodialysis	Adjusted	-	-	-	-	-	-	-	-	-	-	1	1.13	−0.52	2.78	N/A	-
History of minor amputation	Adjusted	-	-	-	-	-	-	-	-	-	-	-	-	-	-	-	-
Hyperlipidemia	Adjusted	-	-	-	-	-	-	-	-	-	-	-	-	-	-	-	-
Hypertension	Adjusted	2	1.07	1.04	1.1	95.8 (0.01)	2	1.08	1.05	1.11	96.3 (0.01)	5	0.35	0.15	0.55	0 (0.44)	**0.001**
Kidney Transplant	Adjusted	2	0.13	0.12	0.15	0 (0.7)	2	0.17	0.01	0.34	82.2 (0.01)	-	-	-	-	-	0.617
Nephropathy	Adjusted	-	-	-	-	-	-	-	-	-	-	2	0.98	0.05	1.91	0 (0.34)	
Neuropathy	Adjusted	-	-	-	-	-	-	-	-	-	-	3	0.8	−0.13	1.72	37.5 (0.2)	
PAD	Adjusted	3	1.04	1.01	1.07	99.8 (0.01)	4	1.08	1.05	1.12	99.6 (0.01)	3	3.49	1.05	5.92	0 (0.64)	**0.025**
PCI	Adjusted	-	-	-	-	-	-	-	-	-	-	-	-	-	-	-	-
Pulmonary disease	Adjusted	-	-	-	-	-	-	-	-	-	-	-	-	-	-	-	-
PVD	Adjusted	-	-	-	-	-	-	-	-	-	-	2	1.82	1.2	2.43	93.7 (0.01)	-
Renal disease	Adjusted	-	-	-	-	-	-	-	-	-	-	-	-	-	-	-	-
Retinopathy	Adjusted	-	-	-	-	-	-	-	-	-	-	-	-	-	-	-	-
Stroke	Adjusted	-	-	-	-	-	1	0.74	0.16	1.32	N/A	1	0.43	−0.89	1.75	N/A	0.673
T1DM	Adjusted	-	-	-	-	-	-	-	-	-	-	-	-	-	-	-	-
T2DM	Adjusted	-	-	-	-	-	-	-	-	-	-	-	-	-	-	-	-

Bold values indicate statistically significant difference based on the amputation level. K: number of studies analyzed in each group; OR: odds ratio; CI: confidence interval; I^2^ (*p*): I-squared statistic measure of heterogeneity (*p* value of heterogeneity); *p*: *p*-value of the difference between examined groups; AKA: above-knee amputation; BKA: below-knee amputation; N/A: not applicable either because of insufficient number of studies (K = 2) or because of insignificant heterogeneity (*p* > 0.05); T2DM: type 2 diabetes mellitus; T1DM: type 1 diabetes mellitus; PVD: peripheral vascular disorder; PCI: percutaneous coronary intervention; PAD: peripheral artery disease; ESRD: end-stage renal disease; EVT: endovascular therapy; DM: diabetes mellitus; CVD: cerebrovascular disease; CPD: chronic pulmonary disease; COPD: chronic obstructive pulmonary disorder; CKD: chronic kidney disease; CAD: coronary artery disease; CABG: coronary artery bypass graft; GI: gastrointestinal.

**Table 6:** A summary of laboratory predictors of major amputation in the adjusted model stratified by the level of amputation.

		AKA					BKA					BKA-AKA					*p*
Predictor	Model	K	OR	Low CI	High CI	I^2^ (*p*)	K	OR	Low CI	High CI	I^2^ (*p*)	K	OR	Low CI	High CI	I^2^ (*p*)	
Albumin	Adjusted	-	-	-	-	-	2	0.56	0.41	0.71	98.3 (0.01)	1	0.61	0.37	0.86	N/A	0.746
ALT	Adjusted	-	-	-	-	-	-	-	-	-	-	-	-	-	-	-	-
Anemia	Adjusted	-	-	-	-	-	-	-	-	-	-	-	-	-	-	-	-
Creatinine	Adjusted	-	-	-	-	-	2	0.73	0.31	1.15	0 (0.63)	1	7.08	−1.95	16.1	N/A	0.169
CRP	Adjusted	-	-	-	-	-	1	1	0.99	1.01	N/A	-	-	-	-	-	-
eGFR	Adjusted	-	-	-	-	-	-	-	-	-	-	-	-	-	-	-	-
ESR	Adjusted	-	-	-	-	-	-	-	-	-	-	-	-	-	-	-	-
Glucose	Adjusted	-	-	-	-	-	1	0.99	0.98	1	N/A	1	1.01	1	1.02	N/A	**0.018**
HbA1C	Adjusted	-	-	-	-	-	-	-	-	-	-	-	-	-	-	-	-
Hemoglobin	Adjusted	-	-	-	-	-	2	1.64	1.6	1.68	99.8 (0.01)	1	0.82	0.54	1.1	N/A	**0.001**
LDL-C	Adjusted	-	-	-	-	-	-	-	-	-	-	-	-	-	-	-	-
Leukocytosis	Adjusted	-	-	-	-	-	2	3.42	1.72	5.13	0 (0.63)	1	8.58	−74.24	91.4	N/A	0.903
Platelet count	Adjusted	-	-	-	-	-	1	2.01	1.99	2.03	N/A	1	0.99	0.99	1	N/A	**0.001**
WBC count	Adjusted	-	-	-	-	-	2	0.92	0.76	1.08	0 (0.6)	1	1.23	1.08	1.38	N/A	**0.006**

Bold values indicate statistically significant difference based on the amputation level. K: number of studies analyzed in each group; OR: odds ratio; CI: confidence interval; I^2^ (*p*): I-squared statistic measure of heterogeneity (*p* value of heterogeneity); *p*: *p*-value of the difference between examined groups; AKA: above-knee amputation; BKA: below-knee amputation; N/A: not applicable either because of insufficient number of studies (K = 2) or because of insignificant heterogeneity (*p* > 0.05); LDL: low density lipoprotein; ESR: erythrocyte sedimentation rate; eGFR: estimated glomerular filtration rate; CRP: c-reactive protein.

**Table 7:** A summary of medication predictors of major amputation in the adjusted model stratified by the level of amputation.

		AKA					BKA					BKA-AKA					*p*
Predictor	Model	K	OR	Low CI	High CI	I^2^ (*p*)	K	OR	Low CI	High CI	I^2^ (*p*)	K	OR	Low CI	High CI	I^2^ (*p*)	
ACE-I	Adjusted	-	-	-	-	-	-	-	-	-	-	-	-	-	-	-	-
Anticoagulants	Adjusted	1	0.94	0.85	1.03	N/A	2	0.98	0.89	1.06	0 (0.92)	-	-	-	-	-	0.528
Antiplatelets	Adjusted	1	0.91	0.88	0.94	N/A	1	0.92	0.89	0.95	N/A	1	1.28	0.83	1.74	N/A	0.37
Beta blocker	Adjusted	-	-	-	-	-	-	-	-	-	-	-	-	-	-	-	-
Statins	Adjusted	1	0.74	0.68	0.81	N/A	1	0.77	0.7	0.83	N/A	2	0.36	0.24	0.48	91.7 (0.01)	**0.001**

Bold values indicate statistically significant difference based on the amputation level. K: number of studies analyzed in each group; OR: odds ratio; CI: confidence interval; I^2^ (*p*): I-squared statistic measure of heterogeneity (*p* value of heterogeneity); *p*: *p*-value of the difference between examined groups; AKA: above-knee amputation; BKA: below-knee amputation; N/A: not applicable either because of an insufficient number of studies (K = 2) or because of insignificant heterogeneity (*p* > 0.05).

**Table 8:** A summary of clinical predictors of major amputation in the adjusted model stratified by the level of amputation.

		AKA					BKA					BKA-AKA					*p*
Predictor	Model	K	OR	Low CI	High CI	I^2^ (*p*)	K	OR	Low CI	High CI	I^2^ (*p*)	K	OR	Low CI	High CI	I^2^ (*p*)	
ABI > 0.8	Unadjusted	1	6.18	0.24	12.12	N/A	1	4.07	0.57	7.58	N/A	1	9.8	−11.4	31	N/A	0.888
ABI < 0.8	Adjusted	1	0.16	−1.84	2.15	N/A	-	-	-	-	-	1	0.94	0.82	1.06	N/A	0.308
CCI ≥ 5	Adjusted	1	1.06	1.02	1.09	N/A	1	1.05	1.01	1.08	N/A	-	-	-	-	-	0.692
Charcot collapse	Adjusted	-	-	-	-	-	-	-	-	-	-	-	-	-	-	-	-
CLI	Adjusted	1	11.9	11.13	12.67	N/A	-	-	-	-	-	2	1.95	1.48	2.41	85.3 (0.01)	**0.001**
DBP	Adjusted	-	-	-	-	-	-	-	-	-	-	-	-	-	-	-	-
Foot ulcer	Adjusted	-	-	-	-	-	-	-	-	-	-	-	-	-	-	-	-
Ischemic wound	Adjusted	-	-	-	-	-	-	-	-	-	-	-	-	-	-	-	-
Laterality (right side)	Adjusted	-	-	-	-	-	-	-	-	-	-	-	-	-	-	-	-
Major tissue loss	Adjusted	-	-	-	-	-	-	-	-	-	-	-	-	-	-	-	-
Non-ambulatory status	Adjusted	-	-	-	-	-	-	-	-	-	-	3	1.71	1	2.42	0 (0.46)	-
Preop gangrene	Adjusted	-	-	-	-	-	-	-	-	-	-	-	-	-	-	-	-
Rutherford ≥ IIb	Adjusted	-	-	-	-	-	-	-	-	-	-	1	3.78	1.34	6.22	N/A	-
SBP	Adjusted	-	-	-	-	-	-	-	-	-	-	-	-	-	-	-	-
Wound dehiscence	Adjusted	-	-	-	-	-	-	-	-	-	-	-	-	-	-	-	-
Wound depth (bone)	Adjusted	-	-	-	-	-	-	-	-	-	-	1	11.67	−35.42	58.76	N/A	-
Wound infection	Adjusted	-	-	-	-	-	-	-	-	-	-	1	3.94	−0.04	7.92	N/A	-

Bold values indicate statistically significant difference based on the amputation level. K: number of studies analyzed in each group; OR: odds ratio; CI: confidence interval; I^2^ (*p*): I-squared statistic measure of heterogeneity (*p* value of heterogeneity); *p*: *p*-value of the difference between examined groups; AKA: above-knee amputation; BKA: below-knee amputation; N/A: not applicable either because of an insufficient number of studies (K = 2) or because of insignificant heterogeneity (*p* > 0.05); SBP: systolic blood pressure; DBP: diastolic blood pressure; CLI: critical limb ischemia; CCI: charlson comorbidity index; ABI: ankle brachial index.

## Data Availability

The original contributions presented in this study are included in the article/Supplementary Materials. Further inquiries can be directed to the corresponding author(s).

## Supplementary Materials

The following supporting information can be downloaded at: https://www.mdpi.com/article/10.3390/healthcare14152340/s1, Table S1: The search strategy employed in our systematic literature search; Table S2: A list of reported predictors of major amputation not eligible for a meta-analysis.

## Abbreviations

ABI	Ankle-Brachial Index
AKA	Above-Knee Amputation
aOR	Adjusted Odds Ratio
BKA	Below-Knee Amputation
BMI	Body Mass Index
CAD	Coronary Artery Disease
CI	Confidence Interval
CLI	Critical Limb Ischemia
COPD	Chronic Obstructive Pulmonary Disease
CRP	C-Reactive Protein
CVD	Cardiovascular Disease
DFU	Diabetic Foot Ulcer
ESRD	End-Stage Renal Disease
I^2^	Higgins’ Inconsistency Statistic
MeSH	Medical Subject Headings
NOS	Newcastle–Ottawa Scale
OR	Odds Ratio
PAD	Peripheral Artery Disease
PRISMA	Preferred Reporting Items for Systematic Reviews and Meta-Analyses
PVD	Peripheral Vascular Disease
RoB	Risk of Bias
STATA	Statistical Software for Data Analysis
WBC	White Blood Cell
