# Supplementary material for: Independent Predictors of Major Limb Amputation: A Systematic Review and Meta-Analysis of Adjusted Risk Factors
Source: Healthcare (Basel). 2026 Aug 1;14(15):2340. doi: 10.3390/healthcare14152340 (PMC13465288; doi:10.3390/healthcare14152340)
Supplement: Supplementary file 1 [file healthcare-14-02340-s001.zip › healthcare-4362088-supplementary.pdf]

Table S1. The search strategy employed in our systematic literature search

Database	No	Search Query	Results
**PubMed**			
	#1	Amputation[tiab] OR amputations[tiab]	48287
	#2	Major[tiab]	1720895
	#3	Knee[tiab] OR elbow[tiab] OR limb[tiab] OR limbs[tiab] OR extremity[tiab] OR extremities[tiab]	539132
	#4	Predict[tiab] OR predictor[tiab] OR predictors[tiab] OR “risk factor”[tiab] OR “risk factors”[tiab] OR “prognostic factor”[tiab] OR “prognostic factors”[tiab] OR regression OR "odds ratio"[tiab]	1735719
	#5	“systematic review”[tiab] OR “meta analysis”[tiab] OR editorial[tiab] OR comment*[tiab] OR meta-analysis[tiab] OR “literature review”[tiab] OR “case report*”[tiab]	1253374
	#6	#1 AND #2 AND #3 AND #4 NOT #6	1028
**Web of Science**			
	#1	AB=Amputation OR AB=amputations	33976
	#2	AB=Major	2507990
	#3	AB=Knee OR AB=elbow OR AB=limb OR AB=limbs OR AB=extremity OR AB=extremities	466159
	#4	AB=Predict OR AB=predictor OR AB=predictors OR AB=“risk factor” OR AB=“risk factors” OR AB=“prognostic factor” OR AB=“prognostic factors” OR TX=regression OR AB="odds ratio"	3384959
	#5	AB=“systematic review” OR AB=“meta analysis” OR AB=editorial OR AB=comment* OR AB=meta-analysis OR AB=“literature review” OR AB=“case report*”	854017
	#6	#1 AND #2 AND #3 AND #4 NOT #6	1000
**Cochrane Registry**			
	#1	Amputation OR amputations	10391
	#2	Major	253469
	#3	Knee OR elbow OR limb OR limbs OR extremity OR extremities	41162
	#4	Predict OR predictor OR predictors OR “risk factor” OR “risk factors” OR “prognostic factor” OR “prognostic factors” OR regression OR "odds ratio"	107493
	#5	“systematic review” OR “meta analysis” OR editorial OR comment* OR meta-analysis OR “literature review” OR “case report*”	98123
	#6	#1 AND #2 AND #3 AND #4 NOT #6	127

**Scopus**			
	#1	TITLE-ABS-KEY (Amputation) OR TITLE-ABS-KEY (amputations)	83746
	#2	TITLE-ABS-KEY (Major)	7424949
	#3	TITLE-ABS-KEY (Knee) OR TITLE-ABS-KEY (elbow) OR TITLE-ABS-KEY (limb) OR TITLE-ABS-KEY (limbs) OR TITLE-ABS-KEY (extremity) OR TITLE-ABS-KEY (extremities)	867703
	#4	TITLE-ABS-KEY (Predict) OR TITLE-ABS-KEY (predictor) OR TITLE-ABS-KEY (predictors) OR TITLE-ABS-KEY (“risk factor”) OR TITLE-ABS-KEY (“risk factors”) OR TITLE-ABS-KEY (“prognostic factor”) OR TITLE-ABS-KEY (“prognostic factors”) OR ALL (regression) OR TITLE-ABS-KEY ("odds ratio")	3748672
	#5	TITLE-ABS-KEY (“systematic review”) OR TITLE-ABS-KEY (“meta analysis”) OR TITLE-ABS-KEY (editorial) OR TITLE-ABS-KEY (comment*) OR TITLE-ABS-KEY (meta-analysis) OR TITLE-ABS-KEY (“literature review”) OR TITLE-ABS-KEY (“case report*”)	5301771
	#6	#1 AND #2 AND #3 AND #4 NOT #6	1028
**Google Scholar**			
	With all of the words	major amputation	-
	With the exact phrase		-
	With at least one of the words	predict risk determinant regression prognostic	-
	Total	Only the first 200 records were selected as per recommendations	200

Table S2. A list of reported predictors of major amputation not eligible for a meta-analysis

			Adjusted Model		
**Author (YOP)**	**Predictor**	**Amputation Level**	**aOR**	**Low CI**	**High CI**
Axley (2020)	ASA (IV)	AKA	9.300	1.900	44.900
	Discharged to Facility	AKA	7.800	2.100	28.300
	Distal embolization	AKA	-	-	-
	OSH Transfer	AKA	17.800	2.700	115.900
	P2Y12 antagonist	AKA	-	-	-
	Preop ambulation w/o assistance	AKA	-	-	-
Cai (2021)	CKD (3A)	BKA	1.320	1.250	1.400
	CKD (3A)	AKA	1.090	1.020	1.170
	CKD (3B)	BKA	1.990	1.840	2.160
	CKD (3B)	AKA	1.410	1.280	1.550
	CKD (4)	BKA	2.720	2.330	3.160
	CKD (4)	AKA	2.180	1.850	2.570
	CLD	BKA	0.920	0.870	0.970
	CLD	AKA	1.010	0.950	1.060
Chen (2015)	TAG	Above the ankle	1.010	1.002	1.020
	Wagner >=4	Above the ankle	21.850	1.625	293.940
	Gram +ve infection	Above the ankle	0.250	0.057	1.140
Chou (2022)	AST	Not defined	-	-	-
	BUN	Not defined	-	-	-
	Cause (pressure vs. trauma)	Not defined	-	-	-
	Depth (tendon/joint)	Not defined	1.090	0.460	2.617
	Height	Not defined	-	-	-
	Location (border)	Not defined	-	-	-
	Location (plantar foot)	Not defined	-	-	-
Clegg (2023)	Ambulatory (assisting device)	BKA-AKA	2.770	1.250	7.280
Elmarsafi (2019)	Delayed healing	BKA-AKA	2.620	1.050	6.490
	Hardware failure	BKA-AKA	1.450	0.510	4.080

	Malunion	BKA-AKA	1.130	0.110	11.580
	Nonunion	BKA-AKA	8.490	2.150	33.510
	Pin tract infection	BKA-AKA	1.970	0.500	7.760
	Postop osteomyelitis	BKA-AKA	2.430	1.010	5.870
	Preop osteomyelitis	BKA-AKA	0.980	0.380	2.540
	Preop soft tissue infection	BKA-AKA	1.170	0.440	3.090
	Recurrent dislocation	BKA-AKA	2.180	0.380	12.390
	Recurrent ulcer	BKA-AKA	0.770	0.280	2.100
	Transfer ulcer	BKA-AKA	2.970	0.620	14.070
Furuyama (2018)	Major defect after debridement	BKA-AKA	6.630	1.290	52.770
	No antiplatelet	BKA-AKA	3.480	1.320	9.670
Genovese (2016)	Renal Failure	Not defined	2.370	0.860	6.570
	Rutherford (<IIb)	Not defined	1.010	0.290	3.580
	Etiology (in-situ thrombosis)	Not defined	1.810	0.770	4.280
	Etiology (thrombosed bypass)	Not defined	3.530	1.590	7.860
	Etiology (Thrombosed stent)	Not defined	1.620	0.580	4.580
	Open Revascularization	Not defined	1.390	0.810	2.390
	Respiratory Failure	Not defined	1.430	0.680	3.040
	Technical Failure	Not defined	6.010	3.360	10.780
Goodney (2010)	Nursing home residence	BKA-AKA	2.900	1.300	6.000
	Two vein segments	BKA-AKA	2.000	1.400	2.800
Hallström (2021)	HDL-C	Not defined	-	-	-
	Albuminuria (macro)	Not defined	-	-	-
	Albuminuria (micro)	Not defined	-	-	-
Hamera (2015)	TAG	BKA-AKA	1.000	0.990	1.102
	CCB use	BKA-AKA	0.310	0.050	1.770
	INR	BKA-AKA	2.490	0.130	46.520
	MPV	BKA-AKA	0.980	0.940	1.030
	Number of implanted stents	BKA-AKA	0.440	0.180	1.070
	Number of patent BTK vessels	BKA-AKA	4.400	0.660	29.200
	Number of patent vessels on foot	BKA-AKA	0.390	0.110	1.370

	Number of prior endovascular procedures	BKA-AKA	0.400	0.190	0.820
	Sum of implanted stent lengths	BKA-AKA	1.000	0.990	1.010
	Target lesion revascularization	BKA-AKA	-	-	-
	TASC (femoropopliteal level)	BKA-AKA	0.950	0.440	2.080
	TLR	BKA-AKA	6.920	1.880	25.480
Higashi (2019)	Revascularization	BKA-AKA	2.920	1.390	6.140
	Sarpogrelate	BKA-AKA	0.990	0.380	2.620
Hüsers (2020)	Perfusion	Not defined	1.790	1.180	2.910
Khan (2021)	Elevated chloride (>106)	Through or above the ankle	-	-	-
	Elevated Creatinine (>120)	Through or above the ankle	-	-	-
	Elevated Potassium (>5.1)	Through or above the ankle	-	-	-
	Elevated urea (>7.1)	Through or above the ankle	1.580	1.050	2.390
	Forefoot lesion	Through or above the ankle	0.120	0.060	0.240
	Midfoot lesion	Through or above the ankle	-	-	-
	Neuroischemic wound	Through or above the ankle	-	-	-
	Neuropathic wound	Through or above the ankle	-	-	-
	Septicemia	Through or above the ankle	2.770	1.410	5.460
Kobayashi (2022)	Heel wound	Not defined	-	-	-
	Pedal arch classification	Not defined	-	-	-
	Toe wound	Not defined	0.290	0.094	0.880
	Visible blood flow to the wound	Not defined	0.430	0.210	0.890
Kow (2019)	Multiple surgery site	BKA	60.320	9.200	395.320
	Disease duration (days)	BKA	1.060	1.023	1.110
	Dxt >=10	BKA	-	-	-
	Monomicrobial	BKA	-	-	-
	Necrotizing fascitis	BKA	-	-	-
	Polymicrobial infection	BKA	-	-	-
Kurniawati (2019)	CRP >5 mg/dl	BKA-AKA	-	-	-
	DM <50 years	BKA-AKA	-	-	-
Long (2020)	History of major amputation	BKA-AKA	3.980	2.620	6.030
	Rest Pain	BKA-AKA	-	-	-

	ARBs	BKA-AKA	-	-	-
	Carotid stenosis	BKA-AKA	-	-	-
	Minor tissue loss	BKA-AKA	-	-	-
	TIA	BKA-AKA	-	-	-
Mendes-Pinto (2021)	Augmentation pressure	AKA	13.030	8.000	18.650
	Pulse wave velocity	AKA	4.710	1.240	8.010
Miyajima (2006)	ASO	BKA-AKA	3.230	1.120	5.110
	Neurological symptoms	BKA-AKA	-	-	-
Mohapatra (2018)	Heel wound	Not defined	3.910	1.840	8.320
Mtshali (2020)	Medical complications	BKA-AKA	1.000	0.450	2.200
Namgoong (2016)	2-hour PPBS (norm)	BKA-AKA	-	-	-
	Creatinine (norm)	BKA-AKA	-	-	-
	CRP (norm)	BKA-AKA	-	-	-
	Cu (norm)	BKA-AKA	-	-	-
	Depth (subcutaneous)	BKA-AKA	-	-	-
	FBS (norm)	BKA-AKA	1.007	1.001	1.013
	Fe (norm)	BKA-AKA	-	-	-
	Ferritin (norm)	BKA-AKA	-	-	-
	Hb (norm)	BKA-AKA	0.640	0.472	0.871
	HDL (norm)	BKA-AKA	-	-	-
	Level (hindfoot)	BKA-AKA	6.150	1.800	20.970
	Location (dorsum)	BKA-AKA	-	-	-
	Monofilament test	BKA-AKA	-	-	-
	TIBC (norm)	BKA-AKA	-	-	-
	WBC (norm)	BKA-AKA	-	-	-
	Zn (norm)	BKA-AKA	-	-	-
Ozbeyaz (2022)	Neutrophil	BKA	0.880	0.740	1.040
	PHR	BKA	1.470	1.106	1.912
Pickwell (2015)	IDSA (3)	Not defined	3.010	1.510	6.010
	IDSA (4)	Not defined	5.400	2.200	13.260
	Increased exudate	Not defined	0.970	0.620	1.530

	Local skin temperature increased	Not defined	1.000	0.640	1.580
	Lymphangitis	Not defined	0.850	0.430	1.680
	Pain on palpation	Not defined	1.360	0.840	2.180
	Periwound edema	Not defined	1.840	1.140	2.980
	Periwound redness in cm (>2)	Not defined	1.490	0.650	3.380
	Periwound redness in cm (0.5-2)	Not defined	1.050	0.450	2.440
	Pretibial edema	Not defined	1.890	1.170	3.050
Polanco-Armenta (2017)	Laterality (left)	Not defined	-	-	-
	Renal Failure	Not defined	-	-	-
	Arrhythmia	Not defined	-	-	-
	ASA >2	Not defined	-	-	-
	Blood transfusion	Not defined	-	-	-
	Drainage tube	Not defined	-	-	-
	LOS >30 day	Not defined	-	-	-
	Osteonecrosis	Not defined	-	-	-
	RA	Not defined	-	-	-
	SAH	Not defined	-	-	-
	Septic arthritis	Not defined	-	-	-
	Skin-skin time >120min	Not defined	-	-	-
Yusof (2015)	DM >10 years	BKA-AKA	-	-	-
Rosedi (2022)	HDL-C (abnormal)	Not defined	2.180	1.210	3.920
	Insulin	Not defined	-	-	-
	LDL-C >2.6	Not defined	-	-	-
	Metformin	Not defined	-	-	-
	Sulphonylurea	Not defined	-	-	-
	TAG >1.7	Not defined	-	-	-
	Underweight	Not defined	2.170	0.780	6.070
Schneider (2018)	uACR (>30)	Above the ankle	-	-	-
	uACR (3-30)	Above the ankle	-	-	-
	ANGPTL2	Above the ankle	-	-	-
	HR	Above the ankle	-	-	-

	Macular edema	Above the ankle	-	-	-
	TNFR1α	Above the ankle	1.290	1.180	1.420
Seo (2023)	MACE	AKA	0.140	0.130	0.150
	MACE	BKA	0.210	0.200	0.220
	MVD	AKA	1.300	1.200	1.370
	MVD	BKA	1.290	1.250	1.320
	RAS inhibitor	AKA	1.040	1.001	1.070
	RAS inhibitor	BKA	1.050	1.010	1.080
	Depression	BKA	-	-	-
	LER	BKA	-	-	-
	PND	BKA	-	-	-
Smith (2016)	Rest Pain	Not defined	-	-	-
	Abnormal wound healing	Not defined	-	-	-
	Ankle pressure <50	Not defined	-	-	-
	Anticoagulant	Not defined	-	-	-
	Antithrombotics	Not defined	-	-	-
	Critical stenosis	Not defined	-	-	-
	Deep wound	Not defined	-	-	-
	Edifoligide	Not defined	-	-	-
	Gangrene (postop)	Not defined	-	-	-
	Graft failure	Not defined	-	-	-
	Graft lesion	Not defined	-	-	-
	History of MI	Not defined	-	-	-
	Inflow lesion	Not defined	-	-	-
	Outflow lesion	Not defined	-	-	-
	QoL (activity)	Not defined	-	-	-
	QoL (Emotional)	Not defined	-	-	-
	QoL (overall)	Not defined	-	-	-
	QoL (Pain)	Not defined	-	-	-
	QoL (social)	Not defined	-	-	-
	QoL (Symptom)	Not defined	-	-	-

	Renal insufficiency (<25)	Not defined	-	-	-
	Renal insufficiency (25-65)	Not defined	-	-	-
	Toe pressure <30	Not defined	-	-	-
	Wound necrosis	Not defined	-	-	-
Soto (2013)	Electrical burn	Above or below the elbow	13.790	6.750	28.160
	Epilepsy	Above or below the elbow	-	-	-
	Impaired consciousness	Above or below the elbow	2.860	1.440	5.700
Spreen (2018)	Impaired renal function	Through or above the ankle	1.480	0.710	3.060
	Rutherford	Through or above the ankle	2.240	1.370	3.670
Taşoğlu (2014)	NLR >=5.2	Not defined	3.100	1.200	7.700
	No arterial back bleeding	Not defined	0.080	0.030	0.200
Torbjörnsson (2020)	Multiple surgery site	Above the ankle	-	-	-
	Open fixation	Above the ankle	-	-	-
	Bedridden	Above the ankle	4.100	2.140	7.860
	Crural/pedal	Above the ankle	-	-	-
	Independent	Above the ankle	-	-	-
	Walking with aid	Above the ankle	1.790	1.270	2.520
	Weelchair-bound	Above the ankle	2.340	1.300	4.190
	Wlfl ischemic grade (1)	Above the ankle	-	-	-
	Wlfl ischemic grade (2)	Above the ankle	-	-	-
	Wlfl ischemic grade (3)	Above the ankle	-	-	-
Zhang (2020)	Compartment pressure	BKA-AKA	0.310	0.130	0.780
	ED	BKA-AKA	-	-	-
	PTT	BKA-AKA	1.010	1.001	1.020
	Time to fasciotomy <6hr	BKA-AKA	1.200	0.380	3.760
	Time to fasciotomy >24hr	BKA-AKA	1.820	0.620	5.400
	White	BKA-AKA	-	-	-

YOP: year of publication; TBI: traumatic brain injury; PTT: partial thromboplastin time; ED: emergency department; QoL: quality of life; AKA: above-knee amputation; BKA: below-knee amputation; MI: myocardial infarction; HR: heart rate; MACE: major adverse cardiovascular event NLR: neutrophil-lymphocyte ratio; PLR: platelet-lymphocyte ratio; RDW: red cell distribution width; LDL: low-density lipoprotein; HDL: high-density lipoprotein; RA: rheumatoid arthritis; LOS: length of hospital stay; DM: diabetes mellitus; WBC: white blood cell; FBS: fasting blood sugar; CRP: C-reactive protein; ASO: anti-streptolysin O; TIA: transient ischemic attack; ARBs: angiotensin receptor blockers.
